# Review of Sunset OC/EC Instrument Measurements During the EPA’s Sunset Carbon Evaluation Project

**DOI:** 10.3390/atmos10050287

**Published:** 2019

**Authors:** Steven Brown, Hilary Minor, Theresa O’Brien, Yousaf Hameed, Brandon Feenstra, Dustin Kuebler, Will Wetherell, Robert Day, Richard Tun, Elizabeth Landis, Joann Rice

**Affiliations:** 1 Sonoma Technology, Inc., 1450 N. McDowell Blvd., Suite 200, Petaluma, CA 94954, USA; 2 Department of Air Quality, Clark County, 4701 W. Russell Rd. Suite 200, Las Vegas, NV 89118, USA; 3 South Coast Air Quality Management District, 21865 Copley Dr. Diamond Bar, CA 91765-4178, USA; 4 Missouri Department of Natural Resources, P.O. Box 176, Jefferson City, MO 65102, USA; 5 Department of Energy and Environment, Government of the District of Columbia, 1200 First Street, N.E. 5th Floor, Washington, DC 20002, USA; 6 U.S. Environmental Protection Agency, Office of Air Quality Planning and Standards, Ambient Air Monitoring Group, Mail Code C304-06, Research Triangle Park, NC 27711, USA

**Keywords:** organic carbon, elemental carbon, black carbon, Sunset OC/EC, Aethalometer, Chemical Speciation Network

## Abstract

To evaluate the feasibility of the Sunset semicontinuous organic and elemental carbon (OC/EC) monitor, the U.S. Environmental Protection Agency (EPA) sponsored the deployment of this monitor at Chemical Speciation Network (CSN) sites with OC and EC measurements via quartz fiber filter collection in Chicago, Illinois; Houston, Texas; Las Vegas, Nevada; St. Louis, Missouri; Rubidoux, California; and Washington, D.C. Houston, St. Louis, and Washington also had collocated Aethalometer black carbon (BC) measurements. Sunset OC generally compared well with the CSN OC (r^2^ = 0.73 across five sites); the Sunset/CSN OC ratio was, on average, 1.06, with a range among sites of 0.96 to 1.12. Sunset thermal EC and CSN EC did not compare as well, with an overall r^2^ of 0.22, in part because 26% of the hourly Sunset EC measurements were below the detection limit. Sunset optical EC had a much better correlation to CSN EC (r^2^ = 0.67 across all sites), with an average Sunset/CSN ratio of 0.90 (range of 0.7 to 1.08). There was also a high correlation of Sunset optical EC with Aethalometer BC (r^2^ = 0.77 across all sites), though with a larger bias (average Sunset/Aethalometer ratio of 0.56). When the Sunset instrument was working well, OC and OptEC data were comparable to CSN OC and EC.

## 1. Introduction

Carbonaceous aerosol is a significant, and often the largest, component of fine particulate matter less than 2.5 microns in diameter (PM2.5) in many areas of the United States. It is composed of organic and elemental carbon (OC, EC) [1], but its composition, sources, and spatiotemporal variations are not well-characterized [2]. OC comprises thousands of individual compounds that can be directly emitted as primary emissions or can be formed in the atmosphere from semivolatile and gaseous precursors over the course of minutes to days. EC is directly emitted from combustion processes, such as from mobile sources or from biomass burning. While it is well-established that elevated PM_2.5_ levels are associated with many health effects, such as respiratory and cardiac disease, the complex interaction of specific health effects from individual compounds or PM_2.5_ components, such as OC and EC, are less well understood.

State and local air monitoring agencies monitor OC and EC in urban areas as part of the national Chemical Speciation Network (CSN), where over 100 samplers across the United States collect filters that are subsequently analyzed for OC and EC on a routine basis. Such measurements have been collected for over 15 years, offering an opportunity to evaluate long-term temporal and spatial trends. As continuous monitoring technology has advanced, the U.S. Environmental Protection Agency (EPA) and other air monitoring agencies have begun to assess whether continuous monitoring technologies could feasibly be used to reduce the frequency and amount of filter-based measurements. If continuous monitors were used to continue the long-term monitoring, they could provide a significant improvement to the data collected in three main ways: (1) provide data every day, rather than on the 1-in-3- or 1-in-6-day schedule typical for filter measurements; (2) provide hourly data, so that data analyses such as source apportionment and diurnal analysis would become feasible; and (3) significantly reduce the cost of sample preparation, shipping, and laboratory analysis. The Sunset OC/EC instrument provides integrated measurements of OC and EC on a customizable sampling time (such as hourly or 2-h intervals) and flow rate (2–9 lpm) via a thermal method similar to that used in CSN, as well as an optical EC (OptEC) measurement that is based on transmission of 660-nm wavelength light through the filter. Summed together, OC and EC provide a measurement of total carbon (TC).

The Sunset OC/EC instrument has been widely used in the United States and throughout the world [3–5]. OC measurements have typically been comparable to other measurements of carbonaceous aerosol, such as from the Aerodyne Aerosol Mass Spectrometer (AMS). At a near-road site in Las Vegas, Nevada, Brown et al. [6] found that AMS-derived OC and Sunset OC were very consistent, with small bias (r^2^ of 0.89, slope of 0.91). In Hong Kong, Lee et al. [7] also found good agreement between Sunset and AMS measurements (r^2^ of 0.87 and slope of 0.88). Other studies had more variation between AMS and Sunset measurements, for example in Riverside (r^2^ of 0.53) [8], Tokyo (r^2^ range of 0.67–0.83 in two seasons) [9], and Pittsburgh (r^2^ of 0.88) [10]. In Riverside, Snyder and Schauer [3] found the Sunset measurements compared well with filter measurements (r^2^ of 0.90 and slope of 1.11). In Atlanta, r^2^ values between the Sunset and the Aerosol Chemical Speciation Monitor (ACSM) were between 0.86 and 0.92 in summer and fall [11].

EC from Sunset and black carbon (BC) from Aethalometer™ instruments have also been compared. In Prague, Zíková et al. [12] found that Sunset OptEC and BC were fairly comparable (within 9%) and had a high correlation with a collocated Thermo Scientific 5012 Multi Angle Absorption Photometer (MAAP) in winter (r^2^ of 0.99 and 0.97 for Sunset OptEC and BC, respectively) and summer (r^2^ of 0.92 and 0.93 for Sunset OptEC and BC, respectively). Thermal EC was not used or reported. In New York, Rattigan et al. [13] found a consistent seasonal difference in BC/EC ratio over the course of three years of measurements, with a ratio of 1.4 in October–March and ratio of 2.0 in April–September. They also found an average OptEC/EC ratio of 0.88 in October–March and 1.04 in April–September. Taken together, this indicates an OptEC/BC ratio of 0.63 during October–March and 0.52 in April–September. Throughout the year, there was a high correlation of BC with EC, with a monthly range of 0.82–0.96. In Ontario, collocated EC and BC measurements also had high correlation (r^2^ of 0.85 and 0.77 at two sites), with a BC/EC ratio of 1.7 at both sites [14]; optical EC measurements were not reported.

To evaluate the utility of the Sunset OC/EC instrument as part of the CSN, the EPA sponsored the deployment of this monitor by local air quality agencies at CSN sites in Chicago, Houston, Las Vegas, St. Louis, Rubidoux, and Washington, D.C. Monitors were operated at these locations, as well as at the EPA in Raleigh, North Carolina, for varying lengths of time during 2012–2017. The primary objectives of the study were to evaluate Sunset instrument performance in various locations and conditions; determine how well the Sunset measurements compare with the CSN and Aethalometer measurements, where available; assess precision and detection limits via injections of a known standard amount of sucrose solution; and determine whether integration of the Sunset OC/EC instrument across a larger number of sites is appropriate for long-term monitoring in the CSN. Results from the study are presented here. The Supplemental Material provides additional statistics comparing the Sunset data to CSN and Aethalometer data, maps of the monitoring sites, and figures showing the ratio between Sunset and CSN or Aethalometer data for all sites, diurnal patterns, and time series of data as they exist in the EPA’s Air Quality System (AQS) and after additional quality control (QC) was done.

## 2. Methods

### 2.1. Monitoring Site Locations

Six locations at existing CSN sites were used in this project: Chicago (Com Ed site in Lawndale, AQS ID 17–031-0076); Houston (Deer Park, AQS ID 48–201-1039); Las Vegas (East Las Vegas, AQS ID 32–003-0540); Rubidoux (Rubidoux, AQS ID 06–065-8001); St. Louis (Blair Street, AQS ID 29–510-0085); and Washington, D.C. (McMillan Reservoir, AQS ID 11–001-0043). Two Sunset instruments were operated at St. Louis from 11 August 2016 through 11 January 2017. Table 1 summarizes the site locations and collection dates of Sunset data. Supplemental Figures show maps for each location. The Chicago site is in southwestern Chicago, in a dense urban neighborhood, approximately 3.7 km southeast of Midway Airport. The Houston site is in a residential neighborhood of eastern Houston, approximately 4.5 km south of industrial facilities in the Houston Ship Channel and 2.6 km east of the Sam Houston Parkway. The Las Vegas site is in east Las Vegas, 1.1 km east of Highway 515 in an urban neighborhood. The Rubidoux site is in a residential neighborhood of Riverside, 150 m southwest of Highway 60. The St. Louis site is north of downtown in an urban residential neighborhood, approximately 250 m west of Interstate 70 and 1.2 km west of the Missouri River, along which are multiple industrial facilities. The Washington, D.C. site is in a northeastern D.C. neighborhood, approximately 1.6 km north of Interstate 395. Sunset, Aethalometer, and CSN data were acquired from the EPA’s AQS in summer 2017.

### 2.2. Sunset OC/EC

In this application, the Sunset OC/EC instrument used a thermal optical method similar to NIOSH 5040 [15–21]. Other methods, such as IMPROVE-A by TOT, could also be used. Aerosol is drawn through a PM_2.5_ cyclone inlet with a carbon denuder and deposited for 47 min at a flow rate of 8 lpm on a quartz fiber filter located in an oven chamber. The collected aerosol is then heated off the filter during an 8-min cycle by heating the filter to 850 °C for 5 min to quantify OC. As the evolved carbon flows through the manganese oxide (MnO_2_) oven, it is converted to carbon dioxide (CO_2_) gas, which is carried in a helium (He) stream and measured directly by a self-contained nondispersive infrared (NDIR) detector system. Next, an oxidizing carrier gas (He with 2% oxygen (O_2_)) is introduced at 850 °C for 3 min to quantify EC, where the EC is detected (similar to the way OC was detected). The remaining 5 min is used for cooling down the oven. During the filter heating, carbonaceous material evolves off the filter as CO_2_, which is quantified using an NDIR detector. EC is determined as any carbon evolved off the filter after the introduction of He/O_2_ once the laser-monitored filter absorbance matches the initial absorbance measured when the filter was first heated. After each hourly analytical cycle, calibration gas of 5% methane (CH_4_) with He flushes the system. Manufacturer-specified detection limits are 0.4 μg C/m^3^ for OC and 0.2 μg C/m^3^ for EC. TC is the sum of OC and EC.

Where reported by the monitoring agency, both thermal EC, referred to as “EC”, and OptEC comparisons are provided here. The OptEC is a measurement of transmittance through the filter at a wavelength of 660 nm prior to the thermal analysis, measuring the amount of absorbance in the sp^2^ bonds of graphitic carbon. Since the measurements of both OptEC from the Sunset and BC from the Aethalometer are based on optical absorbance methods, we compared how consistent measurements from these techniques were to each other and to the thermal EC from CSN. At Chicago, no OptEC was reported. At St. Louis, thermal EC was not reported after 2014 because the instrument needed very frequent filter replacements; this is likely due to high loadings of metal oxides in the ambient PM_2.5_ at the monitoring site. Once only OptEC was measured, the instrument filter did not have to be replaced as often, so only OptEC was reported for the majority of the study.

Two Sunset OC/EC instruments were operated at the EPA site to test instrument setup and quantify bias, precision, and detection limits using injections of a sucrose standard; the equations used to quantify bias, following EPA guidance, are shown below [22]. A known amount (10 μL or 5 μL) of 99.5% sucrose from Sigma Aldrich (product #S9378) was injected into each instrument intermittently over the course of 2 years. The absolute percent difference (*d*) between the observed response from the instrument and the injected amount of carbon was then calculated. The coefficient of variation upper bound (90th percentile) was calculated as the precision estimate:

(1) $$CV=\sqrt{\frac{n\sum_{i=1}^{n}d_{i}^{2}-{\left(\sum_{i=1}^{n}d_{i}\right)}^{2}}{n(n-1)}}\times \sqrt{\frac{n=1}{X^{2}0.1,n-1}}$$

where *X*^2^_0.1,*n*_−_1_ is the highest 10th percentile of a chi-squared distribution with *n* − 1 degrees of freedom. Bias is calculated as the upper bound of the mean absolute value of the percent differences *d* across all *d*_*i*_ values, from the mean of absolute values of all d values (*AB*) and the standard deviation of the absolute values of all d values (*AS*):

(2) $$\text{|}bias\text{|=}AB+t_{\text{0}.\text{95},n\text{−1}}\times \frac{AS}{\sqrt{n}}$$

(3) $$AB=\frac{1}{n}\times \sum_{i=1}^{n}\left|d_{i}\right|$$

(4) $$AS=\sqrt{\frac{n\times \sum_{i=1}^{n}{\left|d_{i}\right|}^{2}-{\left(\sum_{i=1}^{n}\left|d_{i}\right|\right)}^{2}}{n(n-1)}}$$

In addition, the instrument’s response to prebaked, blank quartz fiber filters was used to calculate the detection limit. The detection limit is calculated following 40 Code of Federal Regulations (CFR) part 136, Appendix B [23]:

(5) $$MDL=\bar{X}+t_{\left(n-1,1-\alpha =0.99\right)}\times S$$

where $\bar{X}$ is the mean of replicate method blank results, *t*_(*n*_−_1,1_−_∝=0.99)_ shows the Student’s t value at a 99% confidence level with *n* − 1 degrees of freedom, and *S* is the standard deviation of the blank samples.

### 2.3. CSN URG 3000N Sampler and Lab Analysis

As part of routine measurements in the CSN, quartz fiber filters are prepared and shipped to monitoring sites. Filters are prebaked to remove organic vapor and residue. A URG 3000N sampler is used to collect aerosol on filters, but unlike the Sunset instrument, no denuder is used. Aerosol is sampled at a flow rate of 22 lpm through a PM_2.5_ inlet for 24 h every third or sixth day. OC and EC are then determined via the IMPROVE_A temperature protocol [16] by the Desert Research Institute (DRI) using a DRI Model 2001 carbon analyzer. In this protocol, a 0.5-cm^2^ circular segment of the filter is removed and aerosol are thermally evolved off of the filter (similar to the process for the Sunset instrument), where OC is determined under a nonoxidizing atmosphere with He gas, and then EC is found using a mix of 98% He and 2% O_2_. Carbonaceous aerosol is volatilized off the filter and converted to CO_2_ in an MnO_2_ oxidizer, and then reduced to CH_4_ via a nickel catalyst and quantified as CH_4_ with a flame-ionization detector (FID). For OC, the temperature is ramped to four temperature plateaus at 140 °C, 280 °C, 480 °C, and 580 °C, where the temperature is held constant at each plateau until the response in the FID has returned to baseline for 30 s (i.e., until there is no more carbonaceous material being volatilized from the filter at that temperature). The He/O_2_ atmosphere is then introduced while the temperature is held at 580 °C in order to initially quantify pyrolyzed organic carbon (OP), and then the temperature is increased to 740 °C and 840 °C. The sum of the carbon evolved in the He atmosphere plus the OP is equal to total OC, while the sum of the carbon evolved under the He/O_2_ atmosphere minus the OP is equal to total EC. As reported in the EPA’s 2014 Environmental Technology Verification Report (EPA/600/R-14/308), the precision of this instrument based on replicate analyses is greater than 15% and indicates “a lower degree of data quality than desired”.

### 2.4. Aethalometer

A Magee Scientific Aethalometer was operated at Washington, D.C. (AE21 instrument), St. Louis (AE33 instrument), and Houston (AE21 instrument). The Aethalometer measures BC via an optical method, instead of the thermal method used by the Sunset and CSN [24,25]. Aerosol is sampled through a BGI model sharp cut cyclone (SCC) PM_2.5_ cyclone inlet at 5 lpm and deposited on a filter tape. Every 5 min, the Aethalometer measures the light attenuation at 880 nm through the filter tape, and is converted into a BC concentration by assuming an attenuation cross section of 16.6 m^2^/g. The measured BC is subtracted from the prior measurement of BC to determine the BC collected during the 5 min of sampling. No post-processing of the raw data was done. For example, when the tape on which aerosol is deposited reaches a given saturation point, the tape advances, so that aerosol is now deposited on a new section of tape. When this occurs, there can be an artifact in the data stream that is not automatically accounted for or corrected without post-processing [25–27]. The AE33 has two built-in light sources to automatically correct for this [26], but no correction was made for the AE21 data.

### 2.5. Data Processing and Quality Control

Sunset and Aethalometer data were reported in both local conditions (LC) and standard temperature and pressure (STP). STP data were converted to LC using local meteorological data; all data reported here are in LC. Daily 24-h averages were calculated from hourly Aethalometer and Sunset data where at least 75% of the hourly data were available.

During the project, the agencies operating the Sunset instrument encountered instrument component malfunctions, such as cracked ovens, NDIR detector failure, heating element failure, and pump failure. These issues were not easily diagnosed during operations and led to shifts in baselines and other data issues that made portions of the data unusable for this analysis. The oven and NDIR problems were typically not found early on, since at the time, there was no routine output from the instrument alerting users to these issues or readily available data from CSN for comparison; this resulted in multiple weeks of data being removed prior to analysis. Data were visually inspected on time series to identify periods where there were sudden shifts in concentration, small quantities of data between data gaps, and unusual outliers.

At St. Louis, starting in January 2015, a filter was stuck, and then during March 2015–January 2016, operators suspected contamination, adjusted the thermocouple, and installed a new photodetector. However, the new photodetector was not working correctly, and data did not return to ‘normal’ until after the oven was replaced in January 2016. There were additional issues with keeping the flow steady in June through July 2016. At Washington, D.C., there were periods where OC or EC concentrations were greater than 100 μg/m^3^, even though collocated PM_2.5_ concentrations were low; these data were excluded from analysis here. Prior to May 2014, OC was not reported at this site, so no data were included here for analysis. Only data starting June 2014 were included for analysis, since there were operational issues prior to this time. Data in June 2015 and February–March 2016 were also excluded from analysis because of operational issues associated with a software update in June and a heating coil malfunction at the end of January 2016, which was not fixed until the end of March 2016. Time-series graphics of all measurements at each site and completeness for each parameter are provided in the Supplemental Material.

At Chicago, there was a significant shift in the lowest reported OC values beginning at the end of December 2014, so only data prior to this shift are included here, and only when OC and EC are both reported. Data after January 2015 were excluded from analysis since there was a clear gradual rise in baseline of OC due to degradation of the NDIR. At Houston, there were multiple gaps in the data as NDIR detectors and ovens had to be replaced. Data prior to December 2014 were excluded since older software was used to determine OC/EC and OptEC, the NDIR malfunctioned and was replaced twice, the oven thermocouple malfunctioned and was replaced, there were leaks, and the instrument was sent back to Sunset twice for maintenance. Data during May–August 2015 and July–August 2016 had an unusual shift in OC and EC was near zero; both of these issues occurred when there were leaks in the sampling line, and neither was seen in the collocated CSN measurements.

Data in Las Vegas were intermittent during the course of operations, resulting in many anomalous data points and shifts in data. Only data with multiple weeks of consistent measurements were included for analysis. For example, in November 2012, OC was consistently reported as less than 0.5 μg C/m^3^, and in July and October 2014, the NDIR and heater coils broke and needed to be replaced multiple times; there was vandalism at the site, so the shelter air conditioning unit was not working; and instrument software was not routinely updated. The period of December 2012 to May 2013 was the most consistent and complete period of data and is used here. Given the operational issues at this site, results are not expected to be representative of optimal instrument operations or of other locations, since much of the data were invalidated and due to poor calibration results with sucrose standards, but data that met a minimum quality control are included here for completeness. At Rubidoux, there were two periods where there was a significant shift in the lowest reported OC values (May–September 2014 and March–October 2015), when operators found leaks in the sampling line and the oven had to be replaced twice. These data were screened out from further analysis; time-series graphs showing the data as reported in AQS and after the subsequent QC described above are provided in the Supplemental Material.

This QC process substantially reduced the number of valid Sunset data points compared to the number reported in AQS. Data availability and summary statistics after data processing and validation are available in the Supplemental Material. After QC, there was a range of coincident, collocated 24-hr Sunset and CSN values available for comparison, which is detailed in Table 2. Since there were a number of operational issues throughout the project, the quality of data varies by site. For example, data recovery was low at Las Vegas, Chicago, and Rubidoux, so results for these sites are likely less representative than results for Houston, St. Louis, and Washington, D.C. While these latter three sites also had operational issues—in particular, problems with broken ovens and NDIRs not being detected—sufficient data were collected for comparison to CSN data.

### 2.6. Comparison of Sunset Data to CSN and Aethalometer Data

Detailed measurement quality objectives (MQOs) for comparing Sunset data to CSN and Aethalometer data were discussed in the Project Quality Assurance Project Plan (QAPP) [28]. These MQOs include comparison via linear least squares regression, comparison of means including variability, and ratio of the means. In addition, collocated measurements at St. Louis were used to estimate precision, which is the measure of agreement among repeated measurements of the same property under identical or substantially similar conditions. The MQO for ratio-of-means in CSN measurements was set as 1 ± 0.15, where the coefficient of variation (CV) is used as the measure of variability. We determined if the mean Sunset TC, OC, or EC values over the entire study for each site were comparable to CSN or Aethalometer means by comparing the mean Sunset value to the mean CSN or Aethalometer value ± the CSN or Aethalometer precision. Precision estimates for the CSN URG 3000N instrument are 17.7% for OC, 28.8% for EC, and 15% for TC, and the precision estimate for the Aethalometer is 3.5% for 24-h averaged data [28]. For all statistics, we report values when the two measurements we are comparing occurred on the same day; e.g., for Sunset and CSN OC, only those days with measurements of both, and for Sunset and Aethalometer, only those days with measurements of both. Thus, there will be some differences in reported values, especially when comparing Sunset EC to either Aethalometer BC or CSN EC, since there are many more days with Aethalometer data than with CSN data. In addition to comparing means within the CV, we also report whether concentrations between two measurements were statistically significant based on a Student’s *t*-test.

## 3. Results

### 3.1. Sunset OC Bias and Detection Limit Calculations

Results of CV, bias, and detection limit calculations using data from sucrose injections for the two Sunset OC/EC instruments at the EPA site are shown in Table 3. The CV and bias values meet the data quality objectives of 15%, ranging between 5% to 6% between the two instruments for bias and 6–8% for CV, which is similar to the 8.8% CV across six collocated CSN OC thermal optical reflectance (TOR) measurements [29]. The bias estimates are similar to prior estimates from collocated Sunset OC/EC instrument data, where Bauer et al. [17] estimated bias of 5.3–5.6% for OC. The calculated detection limit for OC was between 1.4 to 1.5 μg/m^3^, which was higher than the estimate of 0.2 μg/m^3^ reported in Bauer et al. [17] and Sciare et al. [30] and higher than the estimated method detection limit (MDL) from CSN of 0.2 μg/m^3^ in Sciare et al. The difference in detection limit calculation methodologies may explain part of the differences among results. The CSN results are calculated as three times the standard deviation of 50 field blanks, while Bauer et al. used a limit of detection calculation as the 95th percentile of the standard deviation across zero air measurements and Sciare et al. took the average value across seven blank filter samples [17,30]. The detection limit found here is similar to an estimated detection limit of 2.0 μg/m^3^ from Zheng et al. [31], who evaluated how results varied under different operational protocols.

### 3.2. Sunset and CSN OC

**Figure 1.**
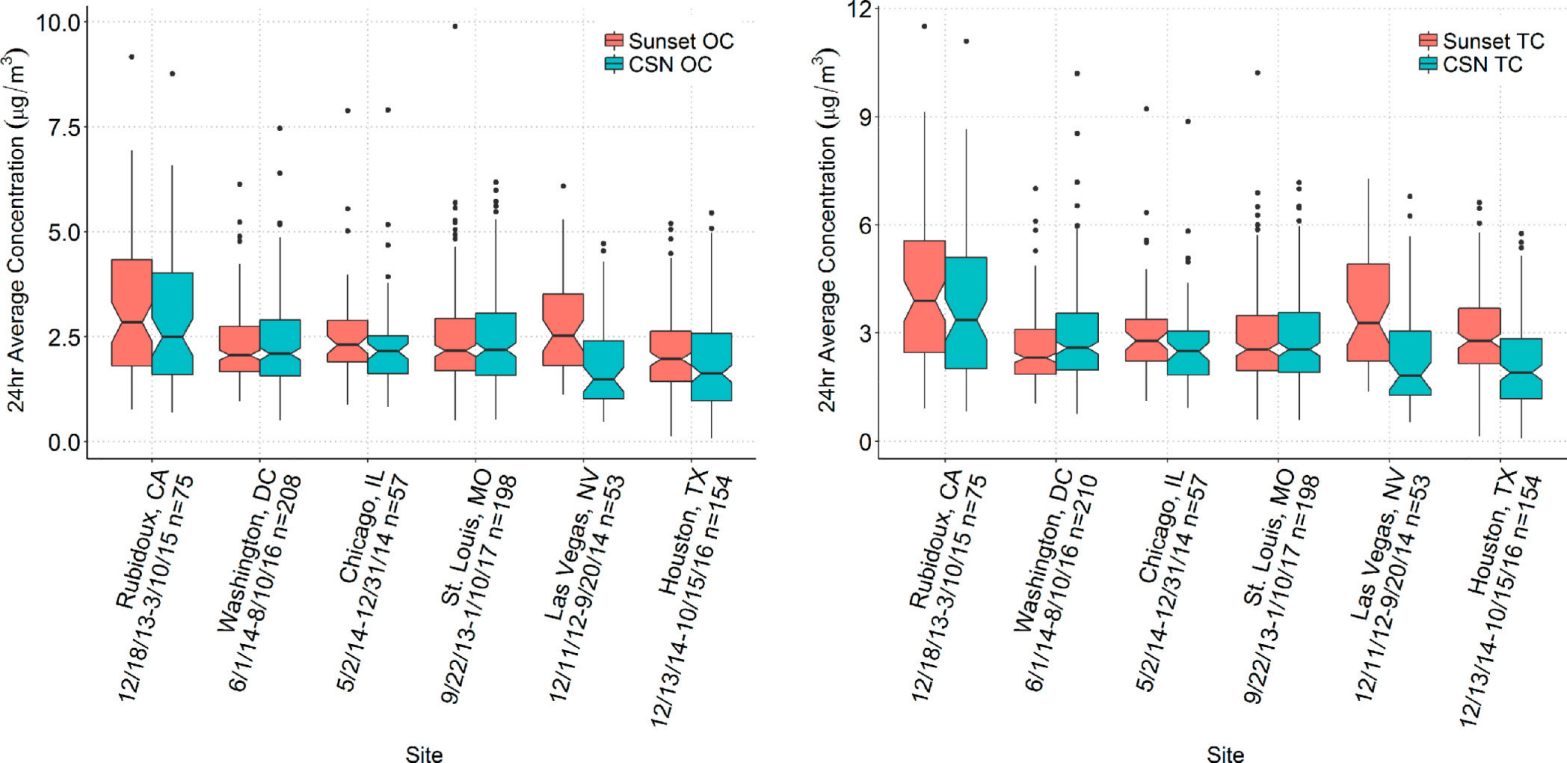
Box plot of Sunset and CSN OC (**left**) and total carbon (TC; **right**) concentrations (μg/m^3^) by site.

**Figure 2.**
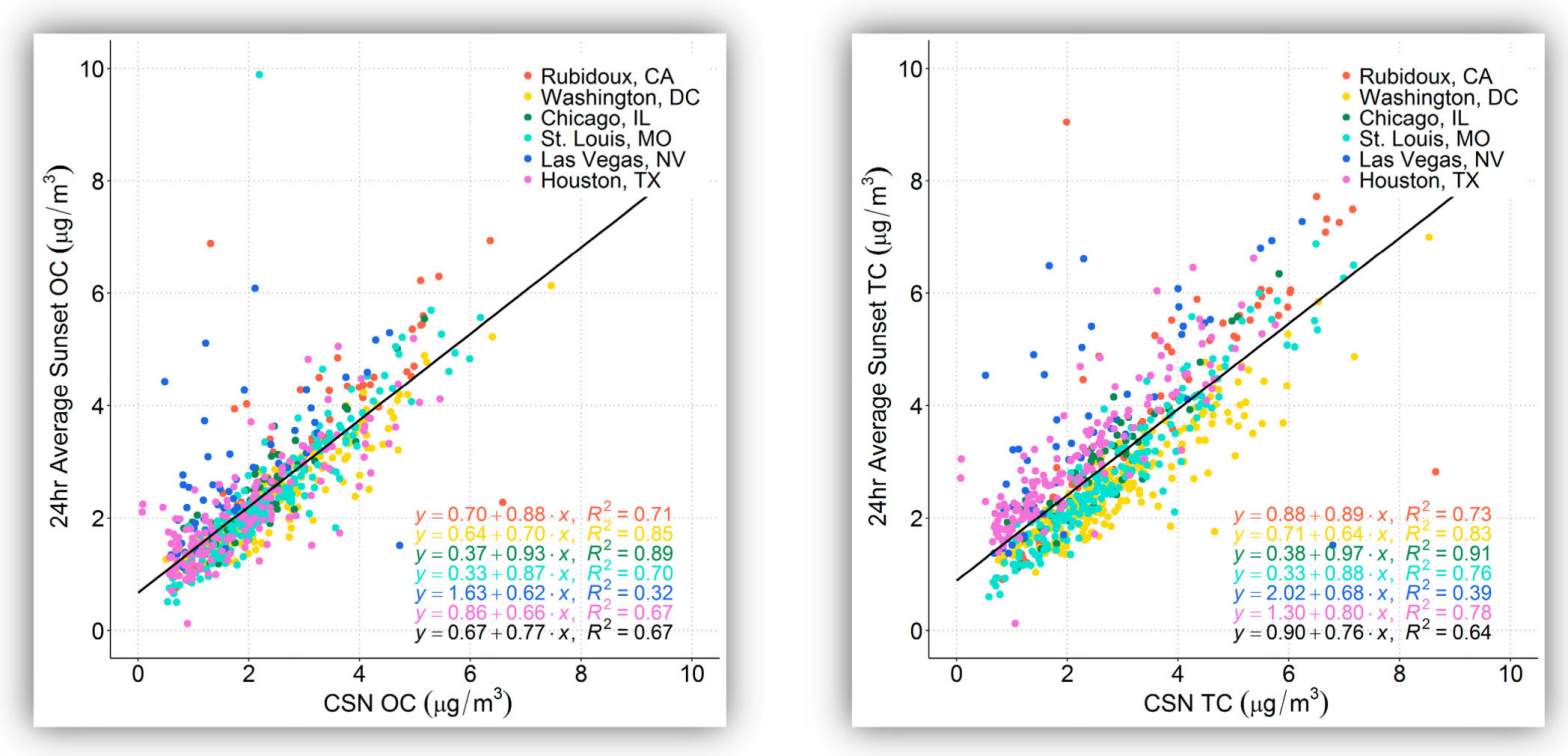
Scatter plot of Sunset and CSN OC concentrations (**left**) and TC concentrations (**right**) (μg/m^3^), colored by site; the linear regression equation written in black is for all data at all sites.

Figure 1 shows box plots of 24-h OC and TC concentrations via Sunset and CSN, and Figure 2 shows Sunset versus CSN data on a scatter plot for both OC and TC. Summary statistics of the Sunset-to-CSN comparison are provided in Table 4 for both OC and EC; only days where both Sunset OC and CSN OC data were available are included. Average Sunset OC concentrations ranged from 2.1 μg/m^3^ at Houston to 3.2 μg/m^3^ at Rubidoux, and average Sunset TC concentrations ranged from 2.6 μg/m^3^ at Washington, D.C. to 4.1 μg/m^3^ at Rubidoux. OC and TC comparisons between Sunset and CSN are similar across sites, so the discussion below focuses on OC. Overall, OC and TC concentrations were higher when measured with the Sunset than in CSN, with an average ratio of means (ROM) of 1.13 for OC and 1.17 for TC. However, this was largely driven by differences between Sunset and CSN at Las Vegas, where the means between the two methods are not comparable. At the other five sites, the ROM for OC was 1.06 and for TC was 1.10, indicating that on average, there was good agreement between the two methods and that MQOs were generally met. As noted earlier, there were significant and frequent operational problems at Las Vegas that likely biased the results there. In addition, at the sites with a larger dataset (e.g., St. Louis and Washington, D.C.), there is little seasonal variation in the Sunset/CSN ratio. When the 8.8% precision (CV) of CSN OC is considered, all sites have comparable means for OC between Sunset and CSN except that at Las Vegas. Where sufficient samples were available, we also found that there was no significant change in the ROM among seasons, i.e., Sunset and CSN means were comparable in all seasons at each site that had at least 10 24-hr values, except at Las Vegas.

The correlation (r^2^) between Sunset and CSN with all measurements was 0.67 and nearly meets the MQO of R = 0.90 if Las Vegas is excluded (r^2^ = 0.73, R = 0.85). The slope is close to 1 at Rubidoux, Chicago, and St. Louis (0.87 to 0.93) and lower at Las Vegas and Houston (0.62 to 0.66). Grouping all measurements together yields a slope of 0.77, with a bias towards Sunset OC being higher than CSN OC. The scatter plot shows a number of outliers, in particular at Las Vegas and Houston, where both CSN and Sunset measurements initially appeared to be valid and were not removed after initial investigation. Without these outliers, the correlation improves marginally, but the bias between the two measurements would remain relatively unchanged. In fact, even with the multiple operational issues that occurred, the bias between Sunset and CSN measurements is fairly consistent across sites.

Overall, the Sunset and CSN OC concentrations compared fairly well across the sites, with an r^2^ of 0.67 and comparable means at all sites except Las Vegas, though with variations in the degree of scatter depending on the frequency of operational issues. There is consistently a bias toward Sunset OC being higher than CSN OC, though this varies by site; however, only at Las Vegas and Houston are the Sunset OC values significantly higher than the CSN OC values, which is likely due to the operational issues encountered at these sites. At St. Louis, where there are nearly 200 measurements included in the analysis, the ratio between Sunset and CSN switches from a Sunset/CSN ratio of 1.06 during the early period of operations of 2013 through early 2015 to 0.91 in 2016 and 2017. The differences between the two periods is that new software, a new oven, and a new NDIR detector were installed, so it is unclear which of these specific actions led to a change in Sunset OC readings. At Las Vegas, there were frequent operational issues and the sample size is relatively small compared to other sites (n = 53), so these results should not be weighted as heavily as those from other sites. With a somewhat broad range of results, Sunset operations likely play a large role in how well the instrument compares to CSN OC data.

In addition, there are differences in how the two thermal–optical methods determine OC and EC; these differences may play a role in how comparable the Sunset (which used NIOSH) and CSN (which used IMPROVE_A) measurements are, even though the total carbon (OC + EC) typically compares well between the two methods [15,16]. A main difference between the two methods is the temperature regime used to determine OC and EC: in NIOSH, the temperature is ramped to 870 °C for determining OC, while in IMPROVE_A, it is ramped to 550 °C. This means that some carbon that is quantified as OC in NIOSH may be quantified as EC in IMPROVE_A; therefore, for a given sample, the NIOSH OC would be higher than the IMPROVE_A OC and the NIOSH EC would be lower than the IMPROVE_A EC. In a direct comparison of these different maximum temperature regimes, Piazzalunga confirmed that a “significant amount” of weakly light-absorbing carbonaceous aerosols were evolved off under 870 °C [32]. In Hong Kong, Wu et al. compared NIOSH and IMPROVE measurements across urban, roadside, and suburban sites over three years and found that differences between the two methods are mostly from the way the OC and EC split is determined. EC from IMPROVE_A was roughly 2.2 times higher than from NIOSH, with more minor differences for OC between the methods [33]. They also found that the amounts of biomass burning and metal oxides such as iron and zinc also impacted how well the two methods compared, where higher metal oxide concentrations led to an increase in the difference between IMPROVE_A and NIOSH EC. Similar results were found in the Southeastern United States during 2003–2005, with total carbon comparable between the two methods but with lower EC from the NIOSH method [34]. TC has similar results to OC, which suggests that disagreement in OC or EC between Sunset and CSN is likely due to how OC and EC are split, as well as other methodological differences, such as the use of the denuder in the Sunset but no denuder with CSN.

### 3.3. Sunset and CSN Thermal EC and Sunset Optical EC

**Figure 3.**
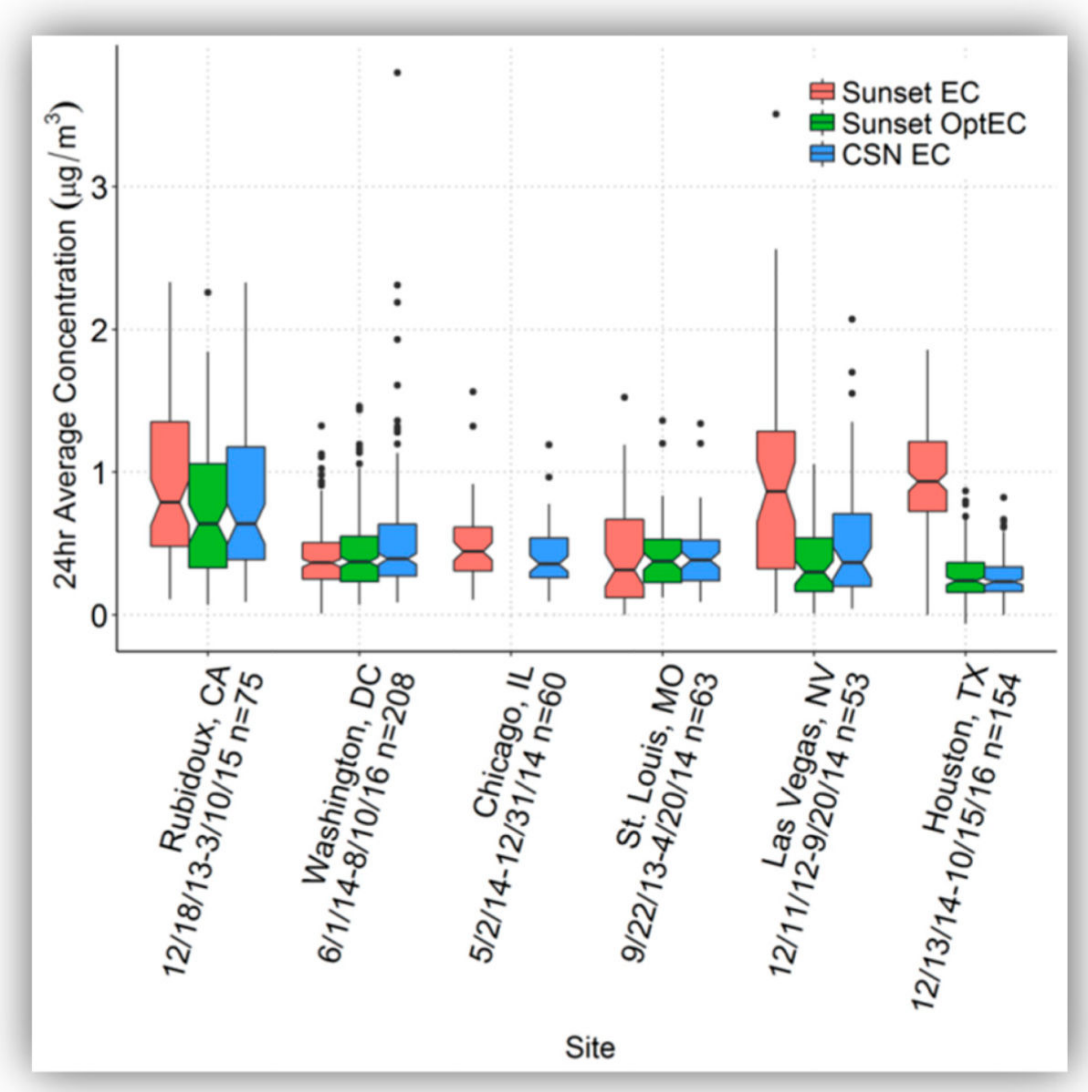
Box plot of Sunset thermal EC, Sunset OptEC, and CSN EC concentrations (μg/m^3^) at each site.

**Figure 4.**
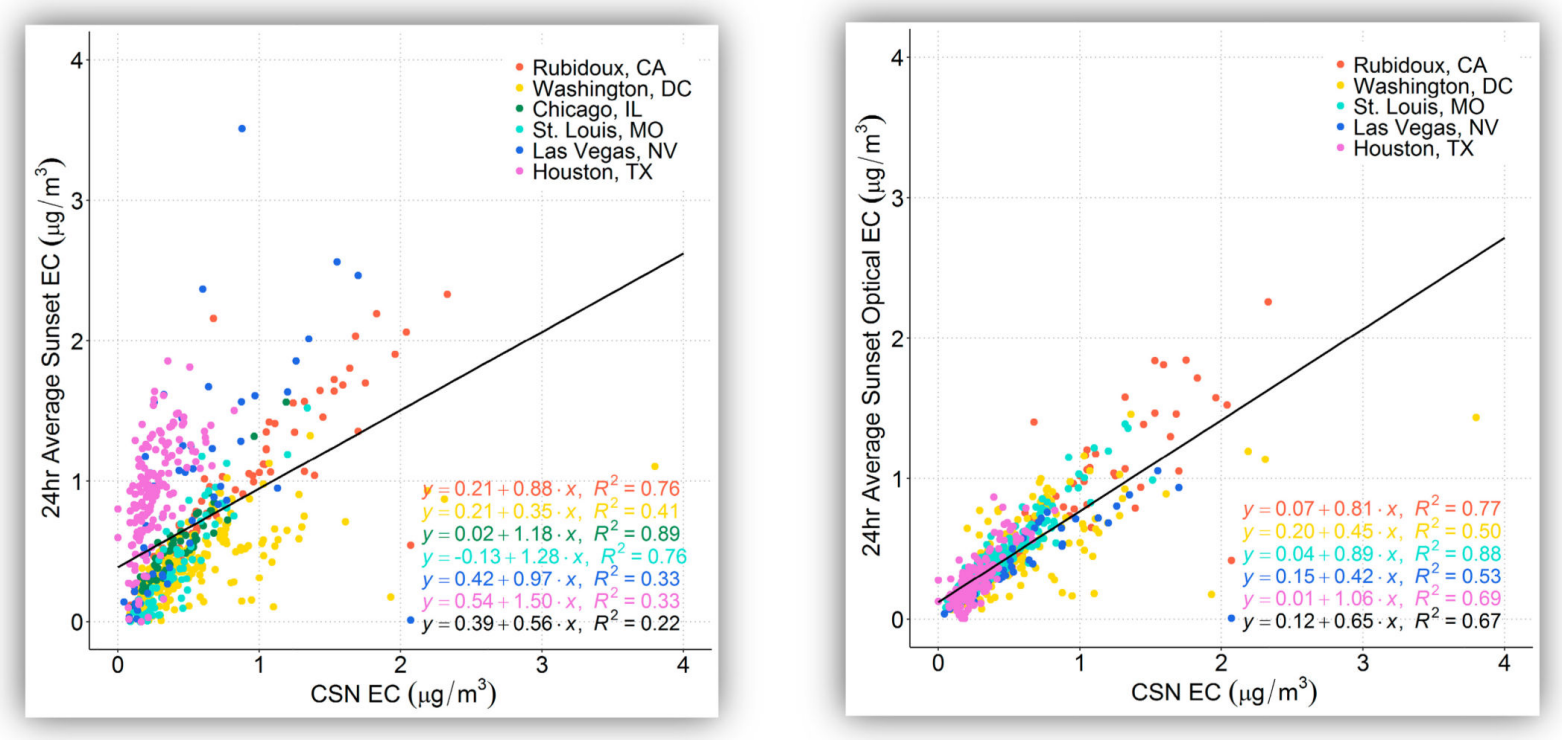
Scatter plot of CSN EC concentrations (μg/m^3^) with Sunset thermal EC (**left**) and Sunset OptEC (**right**), colored by site; the linear regression equation written in black is for all data at all sites.

Summary statistics of the Sunset-to-CSN EC comparison are provided in Table 5; only days where both Sunset EC or OptEC data plus CSN EC were available are included. Here, thermal-derived EC from both Sunset and CSN is referred to simply as “EC”. Figure 3 shows box plots of 24-h EC concentrations at each site via Sunset and CSN, and Figure 4 shows scatter plots comparing CSN EC concentrations with Sunset EC and Sunset OptEC. (For Sunset EC, both thermal EC and optical EC results are shown.) Mean CSN EC concentrations varied between 0.26 μg/m^3^ (Houston) and 0.83 μg/m^3^ (Rubidoux). Sunset thermal EC was similar to CSN EC on average (1.03 ROM) when excluding Las Vegas and Houston; these latter two had much higher ROM (1.76 and 3.55, respectively) and recorded significantly higher Sunset EC compared to CSN EC. OptEC was consistently lower than CSN EC except at Houston; the average Sunset OptEC/CSN EC ratio when excluding Las Vegas and Houston was 0.90. Houston OptEC was much closer to CSN EC than the thermal EC was (ratio of 1.08 instead of 3.55). Thus, except for OptEC at Las Vegas and the thermal EC at Houston, MQOs were met.

There is good agreement between the overall EC means at all sites, with the exception of thermal EC at Las Vegas and Houston, meaning that the ratio of the means between the two measurements are comparable within the precision of each measurement. However, the slope and correlation between Sunset and CSN measurements vary widely. For thermal EC, there is relatively high correlation at St. Louis, Rubidoux, and Chicago (r^2^ of 0.76 to 0.89 for Sunset EC to CSN EC), but there is poor correlation for the other sites (r^2^ of 0.33 to 0.41). Correlations and slopes are more comparable for OptEC to CSN EC, with an r^2^ value of 0.67 when all measurements are pooled together, although with a bias toward CSN EC being higher (slope of 0.65).

Overall, OptEC measurements appear to be more in line with CSN EC than the thermal EC measurements. It is unclear what operational differences occurred at Houston to result in such a large disparity between the site’s EC and OptEC results, which was consistent throughout the study. In addition, having fairly consistent results across all five sites with OptEC, despite numerous operational issues, is significant: the OptEC measurement is fairly consistent when compared to CSN EC despite different locations and operations. The difference between OptEC and thermal EC is partially due to differences in detection limits; 26% of hourly concentrations were below the detection limit of 0.2 μg/m^3^ for thermal EC. Bauer et al. [17] estimated that the detection limit for OptEC is lower than for thermal EC, at between 0.02 and 0.1 μg C/m^3^; having so many of the observations near or below the detection limit for thermal EC likely impacts these results. Potential interferences in the thermal method from metal oxides may also play a role.

### 3.4. Sunset OptEC, Sunset Thermal EC, and Aethalometer BC

**Figure 5.**
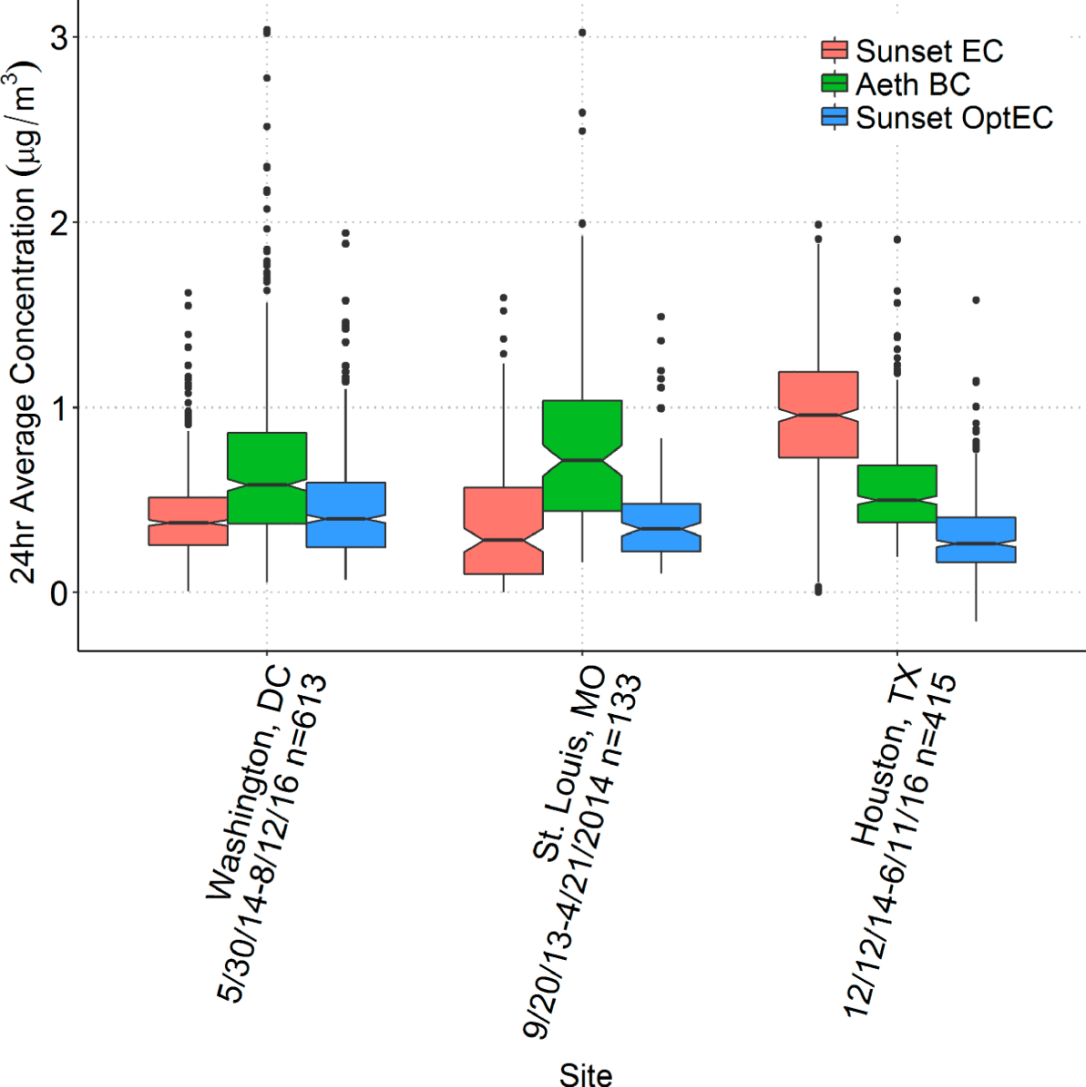
Box plot of 24-h average Sunset OptEC, Sunset EC, and Aethalometer (Aeth) BC concentrations (μg/m^3^).

**Figure 6.**
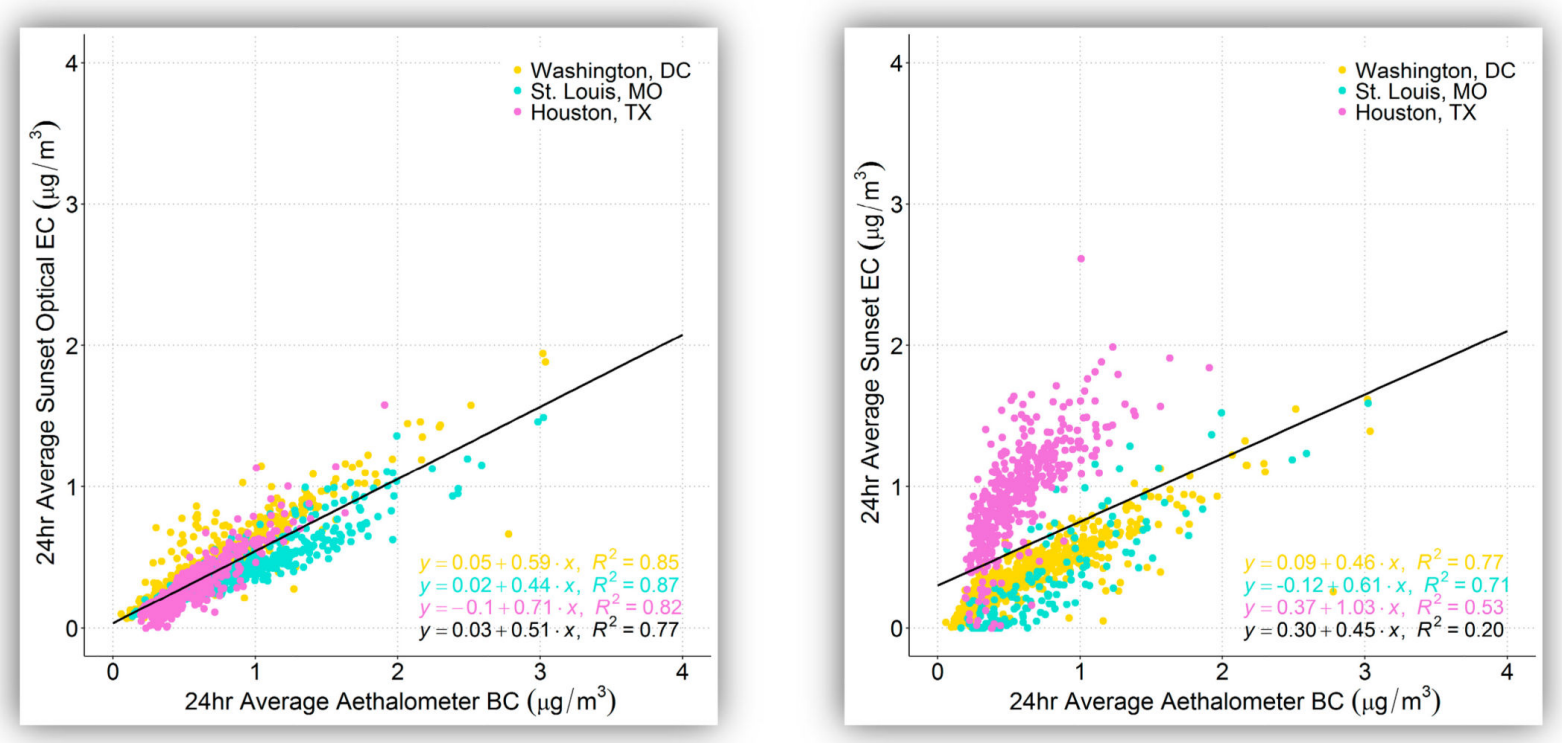
24-h averaged Aethalometer BC compared to Sunset OptEC (left) and Sunset EC (right), colored by site; the linear regression equation written in black is for all data at all sites.

Summary statistics of 24-h Sunset OptEC and Sunset EC to Aethalometer BC are provided in Table 6, and comparisons between the two Sunset measurements and the Aethalometer measurements are shown in Figures 5 and 6. The Sunset OptEC was consistently lower than the Aethalometer BC at all three of the sites that had data for both measurements (Washington, D.C.; St. Louis; and Houston), with a mean ROM of 0.57. This ratio was fairly consistent at each site, with little seasonal variation. For example, the OptEC/BC ratio at Washington, D.C. varied between 0.65 in the winter to 0.70 in the summer. At all three sites, the differences in OptEC and BC measurements were statistically significant and the measurements were not comparable even when accounting for the precision of the Aethalometer (3.5% for 24-h measurements). Sunset EC was consistently lower than BC at Washington, D.C. and at St. Louis, with EC/BC ROMs of 0.60 and 0.46, respectively. Houston, however, was the opposite, with a ROM of 1.68, meaning Sunset EC was consistently higher than BC. As noted earlier, there were intermittent issues with the NDIR and instrumentation that may have been the cause of this result, which is inconsistent with the OptEC results for Houston.

This consistent offset between OptEC and BC is clearly seen in the scatter plot at Figure 6, where the r^2^ value is higher than 0.82 and the intercept is near zero at all three sites. At Washington, D.C. and St. Louis, Sunset EC and BC also have relatively high correlations (r^2^ values of 0.71–0.77). Unlike the results for OptEC at all sites and for EC at the other two sites, Sunset EC and BC have an unusually low correlation and high bias, likely due to operational issues. The relationship between Sunset OptEC and Aethalometer BC is consistent at a range of concentrations, which in this study is up to 2 μg/m^3^ OptEC. However, the slope of the regression and the ratio of the OptEC/BC means varies across sites. At St. Louis, the OptEC/BC ratio is 0.47 and the EC/BC ratio 0.46, while at Washington, D.C., the OptEC/BC ratio is 0.67 and the EC/BC ratio is 0.60, meaning that at these two sites the OptEC and EC are rather consistent when compared with BC. In a multiyear study in New York, similar results were found, with a high correlation between EC and BC and with BC higher by nearly a factor of two during summertime (ratios of 1.3 in winter and 1.8 in summer) [13,35]. In the New York studies, the default absorption coefficient of 16.6 m^2^/g was used, similar to the Aethalometer data collected here; however, they also applied a correction for the nonlinear response in attenuation with increased loading on the filter tape [25,36], which was not done here. They also report variation in the BC/EC ratio that we did not see at the sites in this study, with a higher ratio in summer than in winter. In addition, Rattigan et al. [35] found variation in the BC/EC ratio between the Bronx and Rochester, i.e., between a major urban area and a smaller one. They ascribe part of this variation to changes in optical properties of the ambient aerosol due to emissions from residential wood burning or fuel oil in the wintertime. We do not see the large seasonality in the BC/EC ratio that was observed in Rattigan et al., likely because the New York studies report thermal EC, whereas we report on optical EC here. There is no significant variation in OptEC/BC ratio at Washington, D.C. or Houston (see the Supplemental Material); Houston has a lower OptEC/BC ratio in summer, but there are far fewer days in summer (28) compared to other seasons (81–167) and thus an insufficient sample size to determine a significant difference in summer. At St. Louis, the mean OptEC/BC ratio is significantly lower in spring (0.44) than in fall (0.52), though the range is somewhat small. These consistent seasonal results suggest that absorption efficiency varies seasonally in the same way for both BC and OptEC, while the New York results suggest that the variations in optical absorption of BC likely impact the seasonal results of thermal EC-to-BC ratio. Overall, the OptEC/BC ratio is consistently different at each of the three sites in this study, though generally consistent at any given site across seasons; this is likely due to operational differences among the sites and the sites tested in Rattigan et al. and/or due to differences in aerosol sources between New York and the sites tested in the present study.

### 3.5. Precision from Collocated Measurements in St. Louis

**Figure 7.**
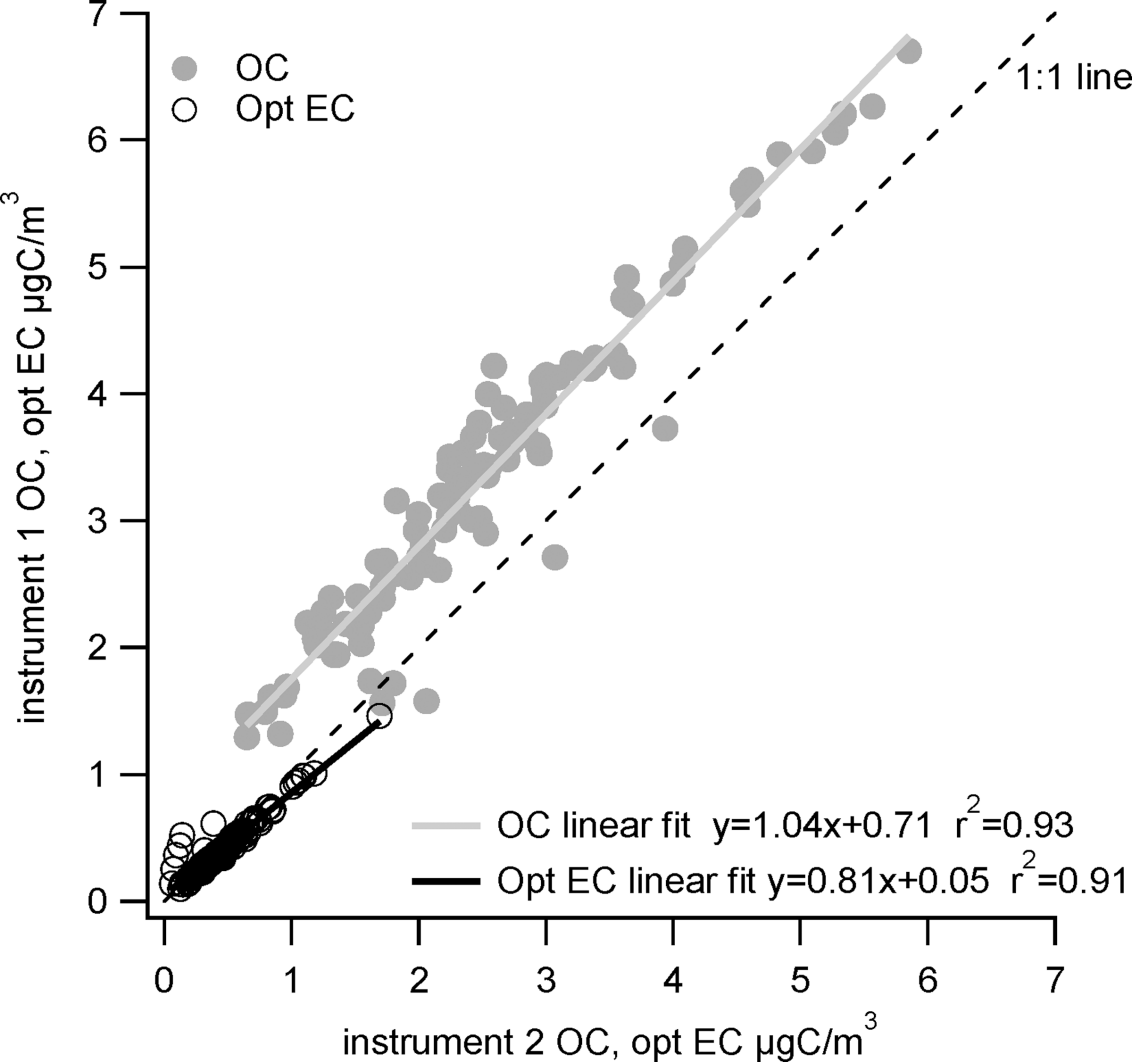
Collocated 24-h OC (gray closed circles) and OptEC (black open circles) measurements at St. Louis from 11 August 2016 through 11 January 2017.

Collocated Sunset instruments were operated at St. Louis during the period from 11 August 2016 through 11 January 2017 and offer a way to gauge precision between two relatively well-operating instruments. A scatter plot of the two instruments is shown in Figure 7 for 24-h average OC and optical EC (n = 102); only OptEC was reported at St. Louis during this time period. Instrument 1, which had been operated during the course of the study, used He as a carrier gas; instrument 2 was set up in the summer of 2016 and used zero air as a carrier gas.

OC concentrations varied between 0.6 and 6.7 μg C/m^3^ and OptEC between 0.1 and 1.7 μg C/m^3^. There is consistent agreement between both OC and OptEC measurements from the two instruments, with r^2^ values of 0.93 for OC and 0.91 for OptEC. There is a bias in the slope (1.04 for OC, 1.12 for OptEC), but with an offset (y-intercept) of 0.7 μg C/m^3^ for OC and no offset for OptEC (y-intercept of zero). This offset for OC may be due to differences in carrier gas, with small impurities in either the He or zero air carrier gas influencing the OC concentrations but not the OptEC. Results are similar to some of the first series of collocated measurements reported by Bauer et al., which found high correlation (0.97 and 0.98) for OC and OptEC when an ambient sample stream was split and routed to two collocated Sunset instruments [17]. They found a lower slope for OptEC (0.82) and magnitude similar to our results for OC (0.95). Their interpretation of results similar to the ones found here at St. Louis was that the instrument produces reliable and reproducible measurements when mass loadings are higher than detection limits. They also note that the instrument needs to be working properly for obtaining such reliable data, similar to the experiences found at multiple sites in this study.

### 3.6. Diurnal Patterns

**Figure 8.**
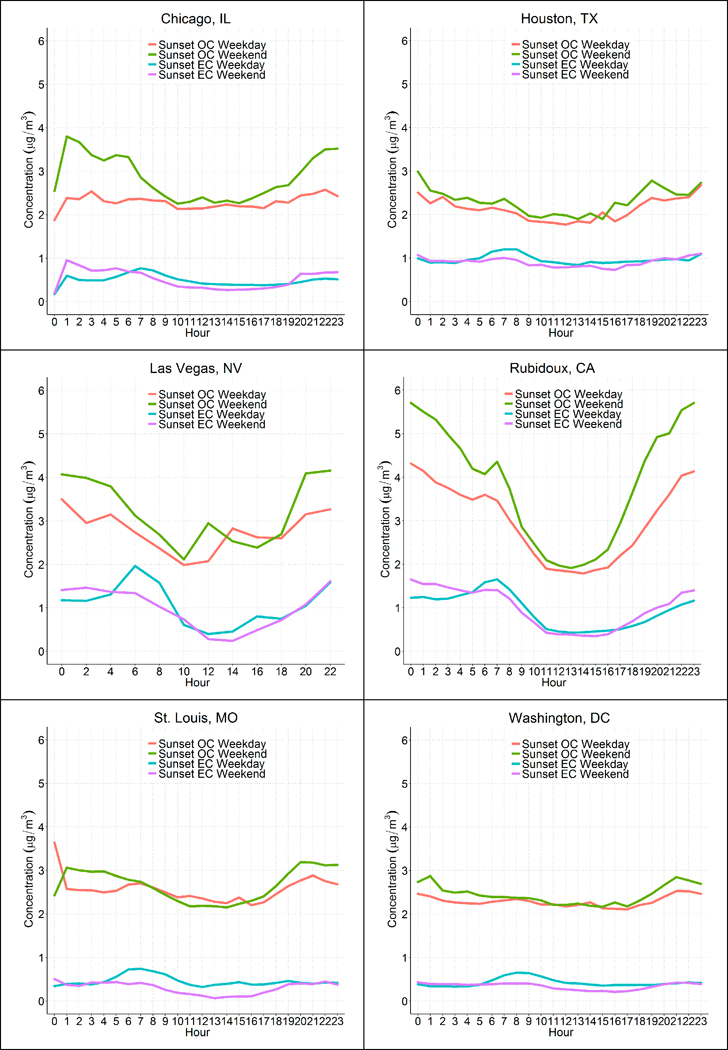
Average hourly Sunset OC and thermal EC concentrations (μg/m^3^) on weekdays and weekends at each site.

A strong diurnal pattern was seen at Rubidoux and Las Vegas for both OC and EC, but the average diurnal pattern at other sites was more muted (Figure 8). At Rubidoux, OC peaked in the evening, while EC peaked in the morning. At the other sites, an overnight peak in OC was also seen, though this overnight peak was only modestly higher than the morning or midday concentrations. EC peaked in the morning at all sites and was clearly higher on weekdays compared to weekends at all sites. OC was slightly higher on weekends compared to weekdays for nearly all hours at each site. This suggests that while ambient EC concentrations may be more driven by changes in traffic in the morning and on weekdays compared to weekends, ambient OC concentrations are a complex mixture of aerosol and semivolatile material that vary due to changes in photochemistry, ambient particulate matter concentration levels, and emissions [2,37,38].

## 4. Conclusions

Sunset OC/EC instruments were operated at six sites collocated with CSN measurements, with Aethalometer measurements also collocated at three of these sites. Operations were quite variable among the sites, with multiple operational issues at all the sites. Critically, when components of the instrument broke, e.g., the oven or NDIR, it was not clear in the data output that there was a problem. This led to multiple weeks to months of operations with a broken component, resulting in large gaps in quality data. Many of these components have since been upgraded or redesigned by Sunset Labs, including changes to the software that will better alert users when a component is damaged.

Despite the operational problems at all sites, Sunset OC and OptEC compared well overall with CSN and Aethalometer measurements, especially at sites that did not have as many operational issues as other sites, such as Rubidoux, St. Louis, and Washington, D.C. Sunset OC was consistently higher than CSN OC, with a Sunset/CSN ratio of 1.06. This ratio is well within the precision of the CSN measurements, so the Sunset and CSN OC are comparable. While on average, the Sunset EC and CSN EC were similar at four of the six sites, there was large scatter and varying biases between these two thermal EC measurements across all sites. This indicates that the thermal EC measurements are not as comparable as the OC measurements are, though part of this may be due to having 26% of Sunset EC measurements below the detection limit. Sunset OptEC data had a much better agreement with CSN EC data, as well as with Aethalometer BC data; Sunset OptEC also has a lower detection limit than Sunset EC, which likely accounts for its improved comparison to CSN EC. Sunset OptEC and thermal EC were consistently lower than the BC, similar to what has been seen previously in the literature, though we did not see seasonal fluctuations in the OptEC/BC ratio. An exception is that EC in Houston was higher than BC, likely due to operational issues that did not impact the OptEC values. That OptEC is quite comparable to both CSN EC and BC indicates that it is a robust and consistent measurement and can be used as an internal validation check the Sunset EC data. There was variation in how Sunset measurements compared to CSN and Aethalometer measurements, but much of the variation is more likely due to differences in operations rather than spatial variability in OC composition or other factors. Overall, with improvements to the NDIR, oven, and software, the Sunset instrument is a viable instrument for field deployment, though as deployed in this study, it is not as well-suited to ‘plug and play’ as other particulate instruments used in routine monitoring networks.

## Supplementary Material

Supplemental Materials

## Figures and Tables

**Table 1.** Summary of measurements by site; date range indicates the time frame when Sunset data were available in the EPA’s Air Quality System (AQS). CSN is Chemical Speciation Network.

City	AQS ID	Site	Operator	Measurements	Dates with Sunset Data
Chicago	17–031-0076	Com Ed, Lawndale	Cook County Dept. of Environmental Control	Sunset, CSN	5/1/14–12/31/15
Houston	48–201-1039	Deer Park	Texas Commission on Environmental Quality (TCEQ)	Sunset, CSN, Aethalometer AE21	8/2/13–12/31/16
Las Vegas	32–003-0540	East Las Vegas	Clark County	Sunset, CSN	8/15/12–12/31/14
Los Angeles	06–065-8001	Rubidoux	South Coast Air Quality Management District	Sunset, CSN	12/17/13–10/14/15
St. Louis	29–210-0085	Blair Street	Missouri Dept. of Natural Resources	Sunset, CSN, Aethalometer AE33	5/7/13–3/30/17
Washington, D.C.	11–001-0043	McMillan Reservoir	District Dept. of the Environment	Sunset, CSN, Aethalometer AE21	10/7/12–8/13/16

**Table 2.** Available collocated 24-h Sunset and CSN organic carbon (OC), elemental carbon (EC) and Sunset optical elemental carbon (OptEC) measurements by site.

Site	No. of Collocated OC Measurements	No. of Collocated EC Measurements	Date Range
Chicago	57	60	5/2/2014–12/31/2014
Houston	154	154 OptEC, 152 EC	12/13/2014–10/15/2016
Las Vegas	53	53	12/11/2012–9/20/2014
Rubidoux	75	75	12/18/2013–3/10/2015
St. Louis	198	198 OptEC, 63 EC	9/22/2013–1/10/2017
Washington, D.C.	208	211 OptEC, 208 EC	6/1/2014–8/10/2016

**Table 3.** Calculations of CV (%), bias (%), and detection limit (μg/m^3^) based on sucrose injection results for two Sunset OC/EC instruments at EPA in Research Triangle Park, North Carolina.

Metric	Sunset 1	Sunset 2
No. of valid measurements	68	85
Coefficient of variance, CV (%)	7.6%	5.8%
Bias	6.3%	5.4%
Detection limit (μg/m^3^)	1.4	1.5

**Table 4.** Summary of Sunset and CSN OC and TC measurements and comparison statistics.

Site	N	Mean Sunset OC	StDev Sunset OC	Mean CSN OC	StDev CSN OC	Ratio of the Means	Comparable Means?	Slope	Confidence Interval	Intercept	r^2^	*p*-Value
Rubidoux	75	3.2	1.7	2.8	1.6	1.12	Yes	0.88	0.74–1.01	0.70	0.71	<0.001
Washington D.C.	208	2.3	0.8	2.4	1.1	0.96	Yes	0.70	0.66–0.74	0.64	0.85	<0.001
Chicago	57	2.5	1.1	2.3	1.2	1.09	Yes	0.93	0.85–1.02	0.37	0.89	<0.001
St. Louis	198	2.4	1.2	2.4	1.1	1.01	Yes	0.87	0.79–0.95	0.33	0.70	<0.001
Las Vegas	53	2.8	1.2	1.8	1.1	1.52	No	0.62	0.37–0.88	1.63	0.32	<0.001
Houston	154	2.1	0.9	1.9	1.2	1.10	Yes	0.66	0.58–0.73	0.86	0.67	<0.001

Site	N	Mean Sunset TC	StDev Sunset TC	Mean CSN TC	StDev CSN TC	Ratio of the Means	Comparable Means?	Slope	Confidence Interval	Intercept	r^2^	*p*-Value

Rubidoux	75	4.1	2.2	3.7	2.1	1.12	No	0.89	0.76–1.01	0.88	0.73	<0.001
Washington D.C.	210	2.6	1.0	2.9	1.4	0.88	No	0.64	0.60–0.68	0.71	0.83	<0.001
Chicago	57	3.0	1.3	2.7	1.3	1.12	No	0.98	0.89–1.06	0.38	0.91	<0.001
St. Louis	198	2.8	1.3	2.8	1.3	1.00	Yes	0.88	0.81–0.95	0.33	0.76	<0.001
Las Vegas	53	3.6	1.7	2.4	1.5	1.53	No	0.68	0.44–0.92	2.02	0.39	<0.001
Houston	154	3.1	1.2	2.2	1.3	1.39	No	0.80	0.73–0.87	1.30	0.78	<0.001

**Table 5.** Summary of Sunset (thermal and optical) and CSN EC measurements and comparison statistics.

Site Name	N	Mean Sunset EC	StDev Sunset EC	Mean CSN EC	StDev CSN EC	Ratio of the Means	Comparable Means?	Slope	Confidence Interval	Intercept	r^2^	*p*-Value

Sunset Thermal EC vs. CSN EC												
Rubidoux, CA	75	0.94	0.57	0.83	0.56	1.13	Yes	0.88	0.77–1.00	0.21	0.76	<0.001
Washington, DC	208	0.40	0.23	0.52	0.42	0.75	Yes	0.35	0.29–0.41	0.21	0.41	<0.001
Chicago, IL	60	0.50	0.26	0.40	0.21	1.24	Yes	1.18	1.07–1.29	0.02	0.89	<0.001
St. Louis, MO	63	0.41	0.35	0.42	0.24	0.99	Yes	1.28	1.10–1.47	−0.13	0.76	<0.001
Las Vegas, NV	53	0.93	0.77	0.53	0.45	1.76	No	0.97	0.58–1.36	0.42	0.33	<0.001
Houston, TX	154	0.93	0.37	0.26	0.14	3.55	No	1.50	1.16–1.84	0.54	0.33	<0.001

Sunset OptEC vs. CSN EC												

Rubidoux, CA	75	0.75	0.52	0.83	0.56	0.90	Yes	0.81	0.71–0.92	0.07	0.77	<0.001
Washington, DC	211	0.44	0.26	0.52	0.42	0.83	Yes	0.45	0.39–0.51	0.20	0.50	<0.001
St. Louis, MO	198	0.42	0.24	0.43	0.25	0.98	Yes	0.89	0.84–0.93	0.04	0.88	<0.001
Las Vegas, NV	53	0.37	0.26	0.53	0.45	0.70	No	0.42	0.31–0.53	0.15	0.53	<0.001
Houston, TX	154	0.28	0.18	0.26	0.14	1.08	Yes	1.06	0.95–1.17	0.01	0.69	<0.001

**Table 6.** Comparison statistics between the Sunset OptEC or Sunset thermal EC and Aethalometer (Aeth) BC measurements.

Site	N	Mean Sunset OptEC	StDev Sunset OptEC	Mean Aeth BC	StDev Aeth BC	Ratio of the Means	Comparable Means?	Slope	Confidence Interval	Intercept	r^2^	*p*-Value
Washington D.C.	618	0.45	0.27	0.67	0.43	0.67	No	0.59	0.57–0.61	0.05	0.85	<0.001
St. Louis	544	0.40	0.22	0.85	0.47	0.47	No	0.44	0.43–0.46	0.02	0.87	<0.001
Houston	415	0.31	0.20	0.57	0.26	0.54	No	0.71	0.68–0.75	−0.10	0.82	<0.001

Site	N	Mean Sunset Thermal EC	StDev Sunset Thermal EC	Mean Aeth BC	StDev Aeth BC	Ratio of the Means	Comparable Means?	Slope	Confidence Interval	Intercept	r^2^	*p*-Value

Washington D.C.	613	0.40	0.23	0.67	0.43	0.60	No	0.46	0.44–0.48	0.09	0.77	<0.001
St. Louis	133	0.38	0.36	0.82	0.50	0.46	No	0.61	0.54–0.68	−0.12	0.71	<0.001
Houston	415	0.95	0.37	0.57	0.26	1.68	No	1.03	0.93–1.12	0.37	0.53	<0.001
