# Supplementary material for: Review of Sunset OC/EC Instrument Measurements During the EPA’s Sunset Carbon Evaluation Project
Source: Atmosphere (Basel). Author manuscript; Available in PMC 2020 Jul 23. (PMC7376833; doi:10.3390/atmos10050287)
Supplement: Supplemental Materials [file NIHMS1599799-supplement-Supplemental_Materials.pdf]

Supplementary Information

- Summary statistics of parameter by site
- Maps of monitoring site locations
- Time series of Sunset data available in AQS by site, annotated where data were excluded from the analysis presented here
- Box plots of Sunset and Aethalometer data by hour by site
- OptEC/BC ratio box plots by season and site

Table S-1. Summary of 24-hr average valid data by site and variable. Completeness percentage is calculated for Sunset data only; SD is standard deviation.

Site Name	Variable	Count	Mean	Min	Max	Sd	Start Date	End Date	Expected Count	Completeness
Rubidoux, CA	CSN EC	469	0.7	0.0	3.0	0.5	8/1/2012	7/29/2016	1458	
Rubidoux, CA	CSN OC	469	2.7	0.4	9.5	1.3	8/1/2012	7/29/2016	1458	
Rubidoux, CA	Sunset EC	237	1.0	0.1	4.1	0.6	12/17/2013	10/14/2015	666	32%
Rubidoux, CA	Sunset OC	237	3.3	0.7	15.8	1.9	12/17/2013	10/14/2015	666	32%
Rubidoux, CA	Sunset OptEC	237	0.8	0.1	3.5	0.6	12/17/2013	10/14/2015	666	36%
Washington, DC	Aeth BC	1466	0.7	0.1	3.4	0.4	8/1/2012	12/22/2016	1604	
Washington, DC	CSN EC	544	0.5	0.0	3.8	0.3	8/4/2012	3/29/2017	1698	
Washington, DC	CSN OC	544	2.4	0.3	10.4	1.3	8/4/2012	3/29/2017	1698	
Washington, DC	Sunset EC	644	0.4	0.0	1.6	0.2	10/7/2012	8/13/2016	1406	32%
Washington, DC	Sunset OC	644	2.3	1.0	7.1	0.9	8/20/2013	8/13/2016	1089	32%
Washington, DC	Sunset OptEC	649	0.4	0.1	1.9	0.3	5/28/2013	8/13/2016	1173	46%
Washington, DC	Sunset TC	647	2.6	1.0	8.3	1.0	1/1/2013	8/13/2016	1320	59%
Chicago, IL	CSN EC	429	0.4	0.0	1.9	0.2	8/1/2012	7/29/2016	1458	
Chicago, IL	CSN OC	429	2.3	0.1	10.5	1.2	8/1/2012	7/29/2016	1458	
Chicago, IL	Sunset EC	191	0.5	0.1	1.6	0.3	5/1/2014	12/31/2015	609	29%
Chicago, IL	Sunset OC	181	2.5	0.6	7.9	1.1	5/1/2014	12/31/2015	609	29%
Chicago, IL	Sunset TC	182	3.0	0.7	9.2	1.3	5/1/2014	12/31/2015	609	31%
St. Louis, MO	Aeth BC	1501	0.8	0.1	4.7	0.5	8/1/2012	3/30/2017	1702	
St. Louis, MO	CSN EC	539	0.4	0.0	1.5	0.2	8/1/2012	3/29/2017	1701	
St. Louis, MO	CSN OC	539	2.5	0.5	9.7	1.2	8/1/2012	3/29/2017	1701	
St. Louis, MO	Sunset EC	202	0.4	0.0	1.6	0.4	5/7/2013	4/22/2014	350	32%
St. Louis, MO	Sunset OC	658	2.4	0.5	9.9	1.1	5/7/2013	3/30/2017	1423	32%
St. Louis, MO	Sunset OptEC	658	0.4	0.1	1.5	0.2	1/1/2013	3/30/2017	1549	58%
St. Louis, MO	Sunset TC	658	2.8	0.6	10.2	1.2	1/1/2013	3/30/2017	1549	46%
Las Vegas, NV	CSN EC	378	0.6	0.0	2.9	0.6	8/1/2012	7/26/2016	1455	
Las Vegas, NV	CSN OC	378	2.3	0.0	9.8	1.6	8/1/2012	7/26/2016	1455	

Site Name	Variable	Count	Mean	Min	Max	Sd	Start Date	End Date	Expected Count	Completeness
Las Vegas, NV	Sunset EC	207	1.1	0.0	7.6	1.1	8/15/2012	12/31/2014	868	26%
Las Vegas, NV	Sunset OC	211	2.9	1.1	12.6	1.5	8/15/2012	12/31/2014	868	26%
Las Vegas, NV	Sunset OptEC	210	0.4	0.0	1.7	0.3	8/15/2012	12/31/2014	868	24%
Las Vegas, NV	Sunset TC	211	3.9	1.2	14.6	2.2	8/15/2012	12/31/2014	868	24%
Houston, TX	Aeth BC	1398	0.5	0.1	1.9	0.2	8/1/2012	6/11/2016	1410	
Houston, TX	CSN EC	547	0.3	0.0	0.9	0.1	8/1/2012	3/29/2017	1701	
Houston, TX	CSN OC	547	1.9	0.0	10.4	1.3	8/1/2012	3/29/2017	1701	
Houston, TX	Sunset EC	697	0.7	0.0	2.6	0.5	8/1/2013	12/31/2016	1248	32%
Houston, TX	Sunset OC	697	2.4	0.1	8.2	1.1	8/1/2013	12/31/2016	1248	32%
Houston, TX	Sunset OptEC	696	0.3	-0.2	1.6	0.2	8/2/2013	12/31/2016	1247	56%
Houston, TX	Sunset TC	697	3.1	0.1	10.6	1.3	8/1/2013	12/31/2016	1248	56%

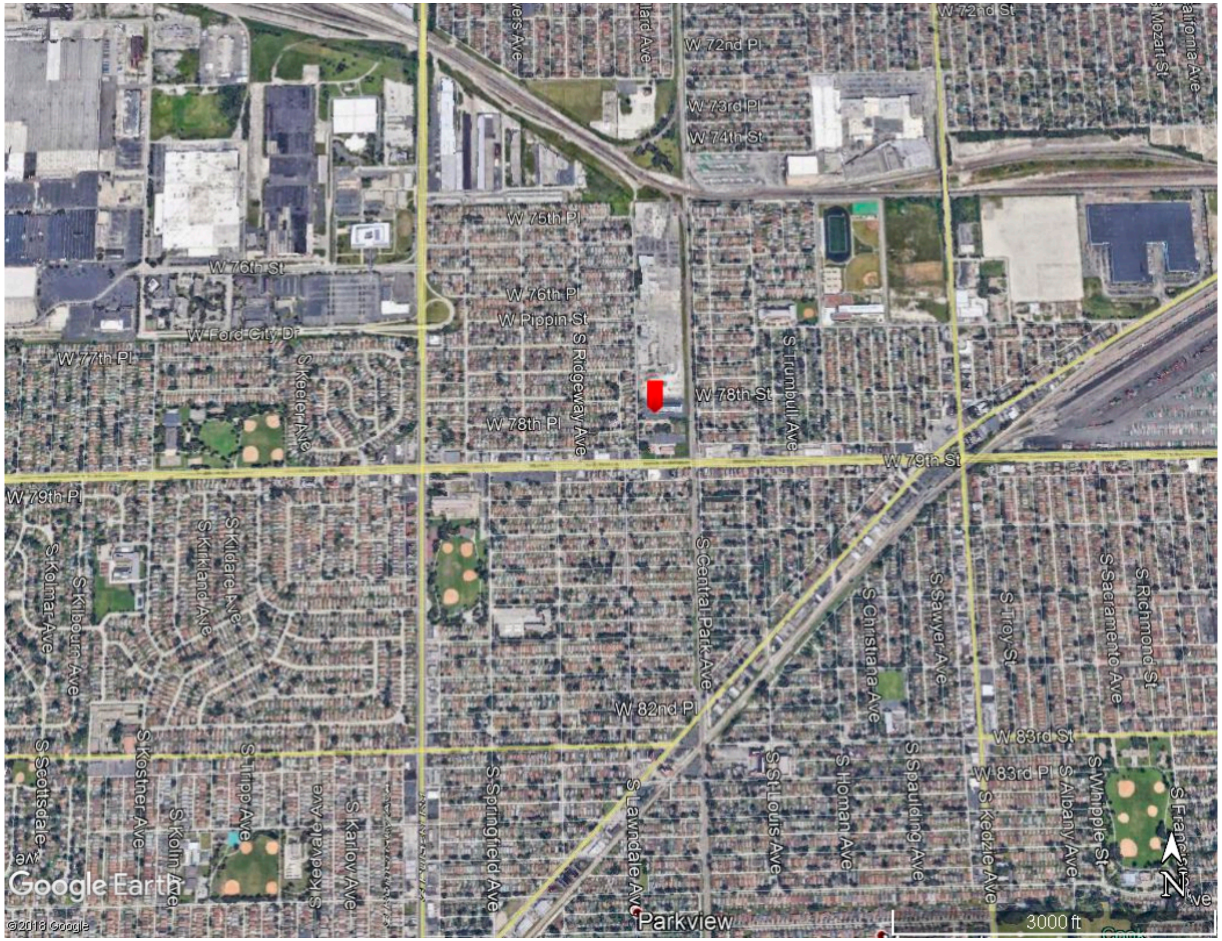

Figure S-1. Satellite view from Google Earth of Chicago Com Ed monitoring site AQS ID 17-031-0076 (red marker).

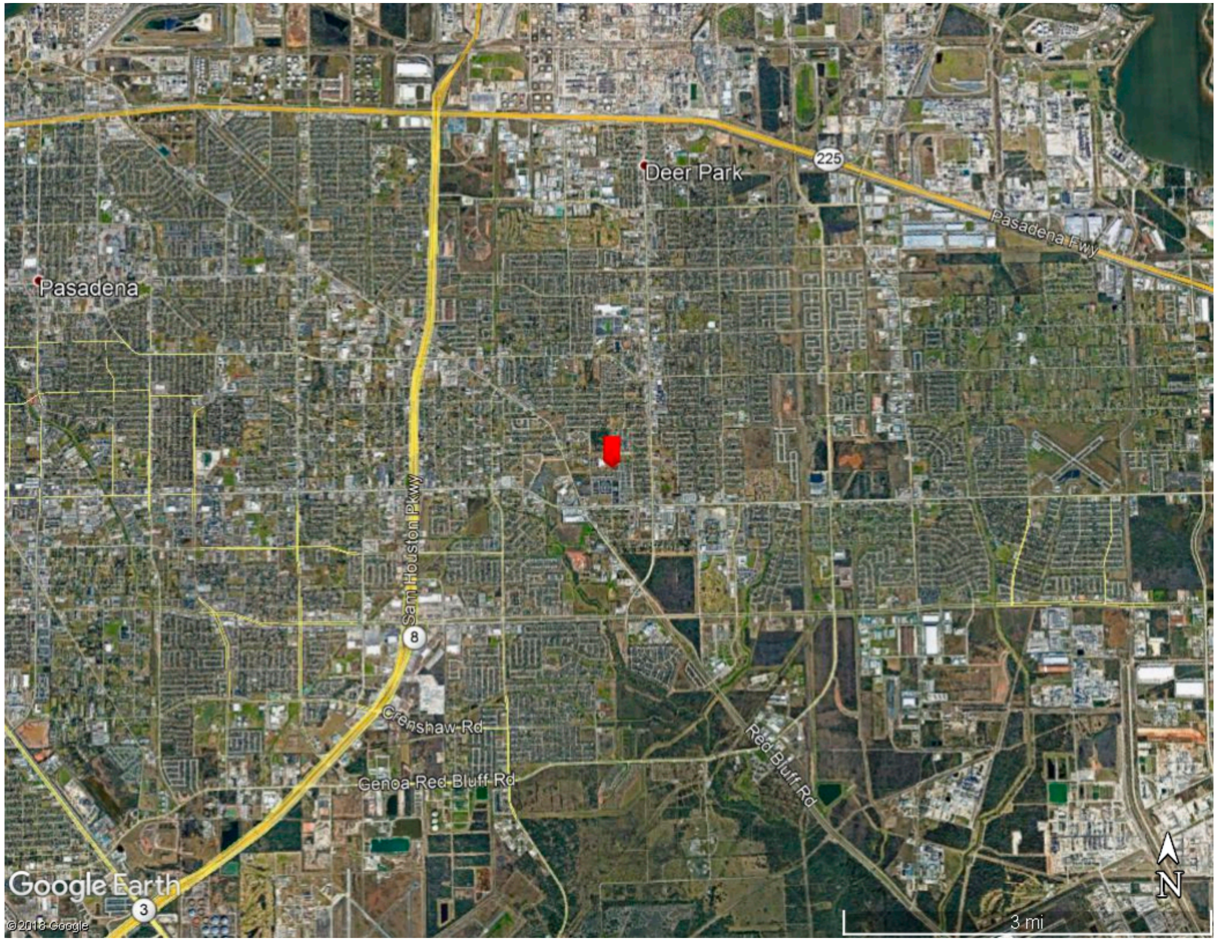

Figure S-2. Satellite view from Google Earth of Houston Deer Park monitoring site AQS ID 48-201-1039 (red marker).

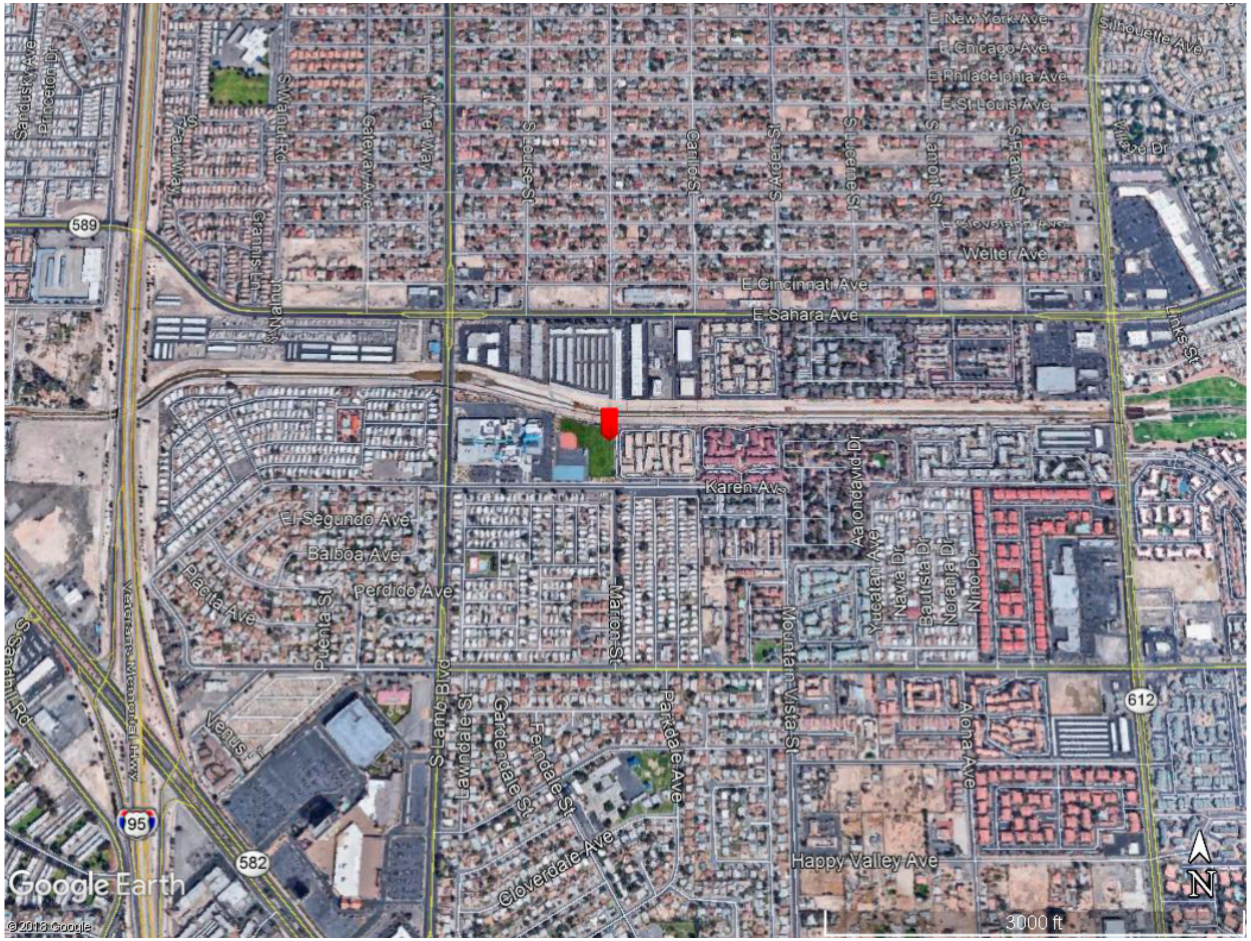

Figure S-3. Satellite view from Google Earth of East Las Vegas monitoring site AQS ID 32-003-0540 (red marker).

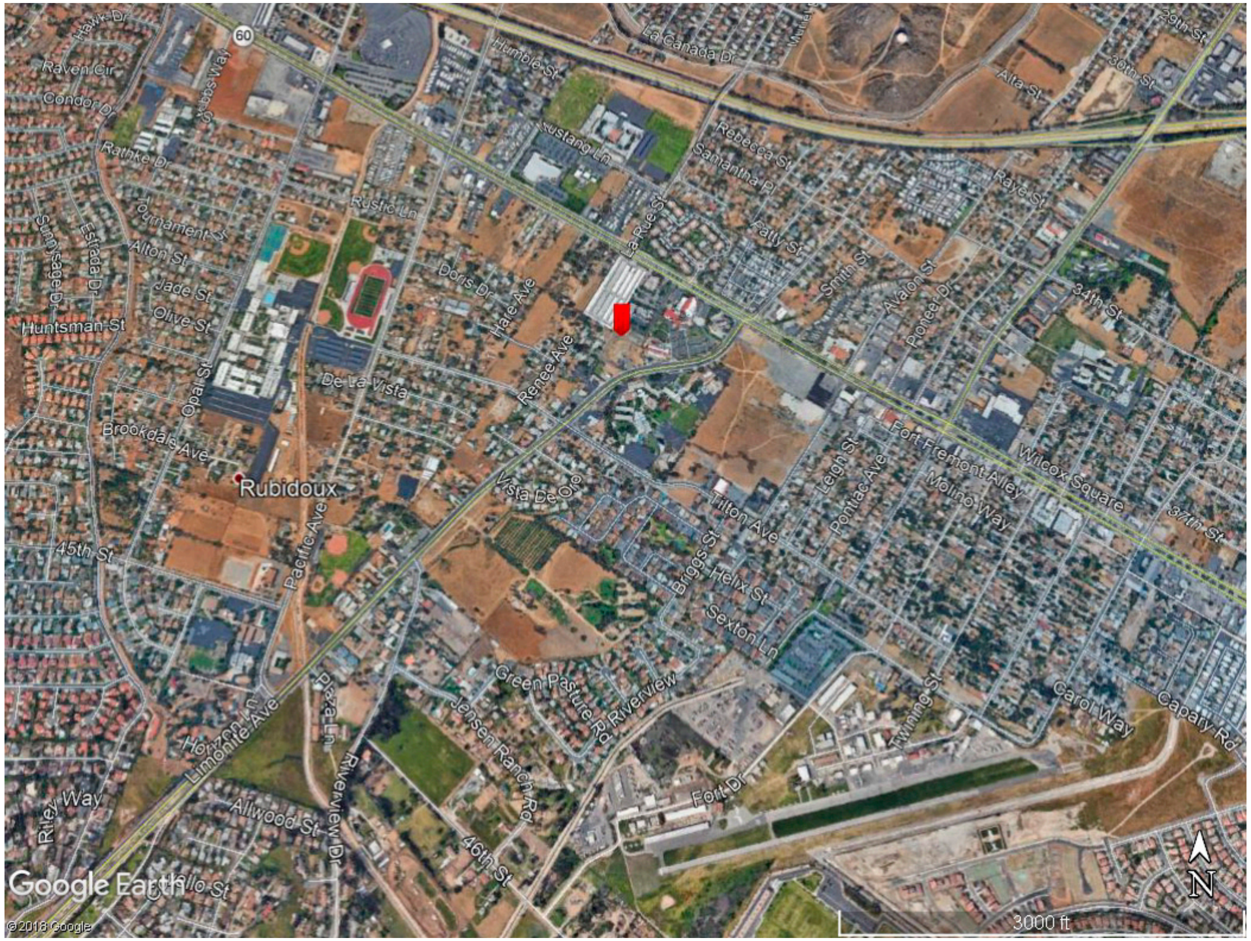

Figure S-4. Satellite view from Google Earth of Rubidoux monitoring site AQS ID 06-065-8001 (red marker).

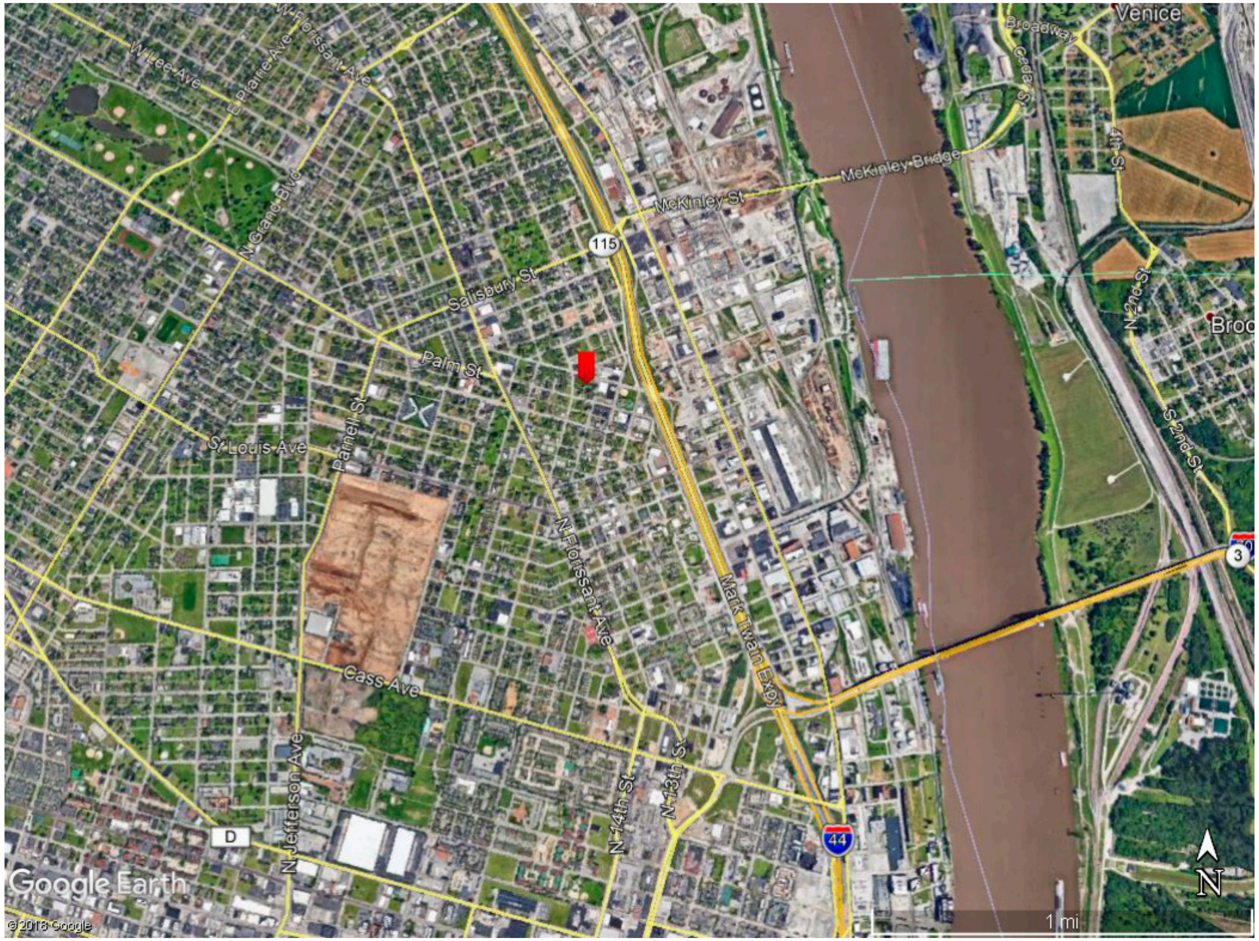

Figure S-5. Satellite view from Google Earth of St. Louis Blair St. monitoring site AQS ID 29-510-0085 (red marker).

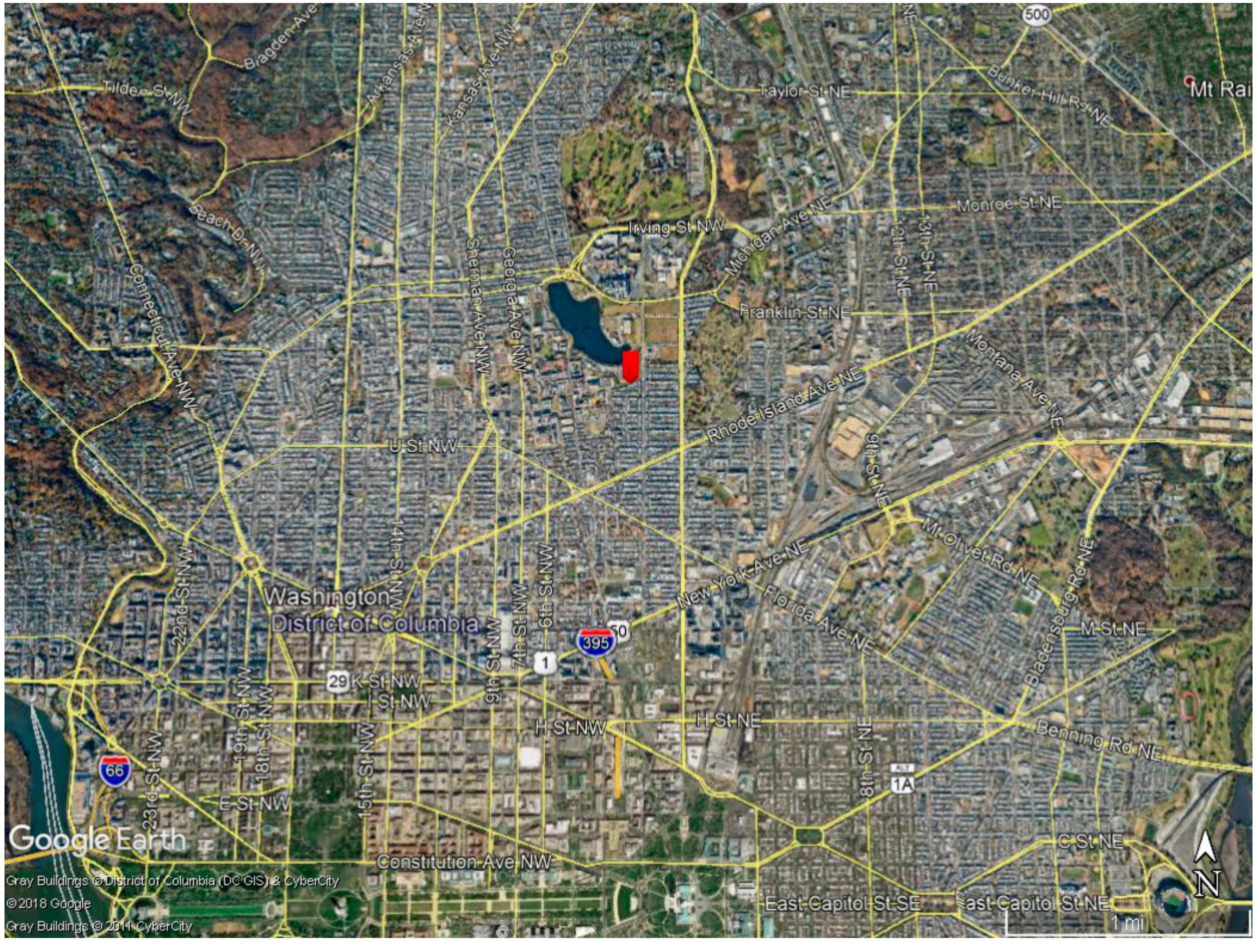

Figure S-6. Satellite view from Google Earth of Washington, D.C. McMillan Reservoir monitoring site AQS ID 11-001-0043 (red marker).

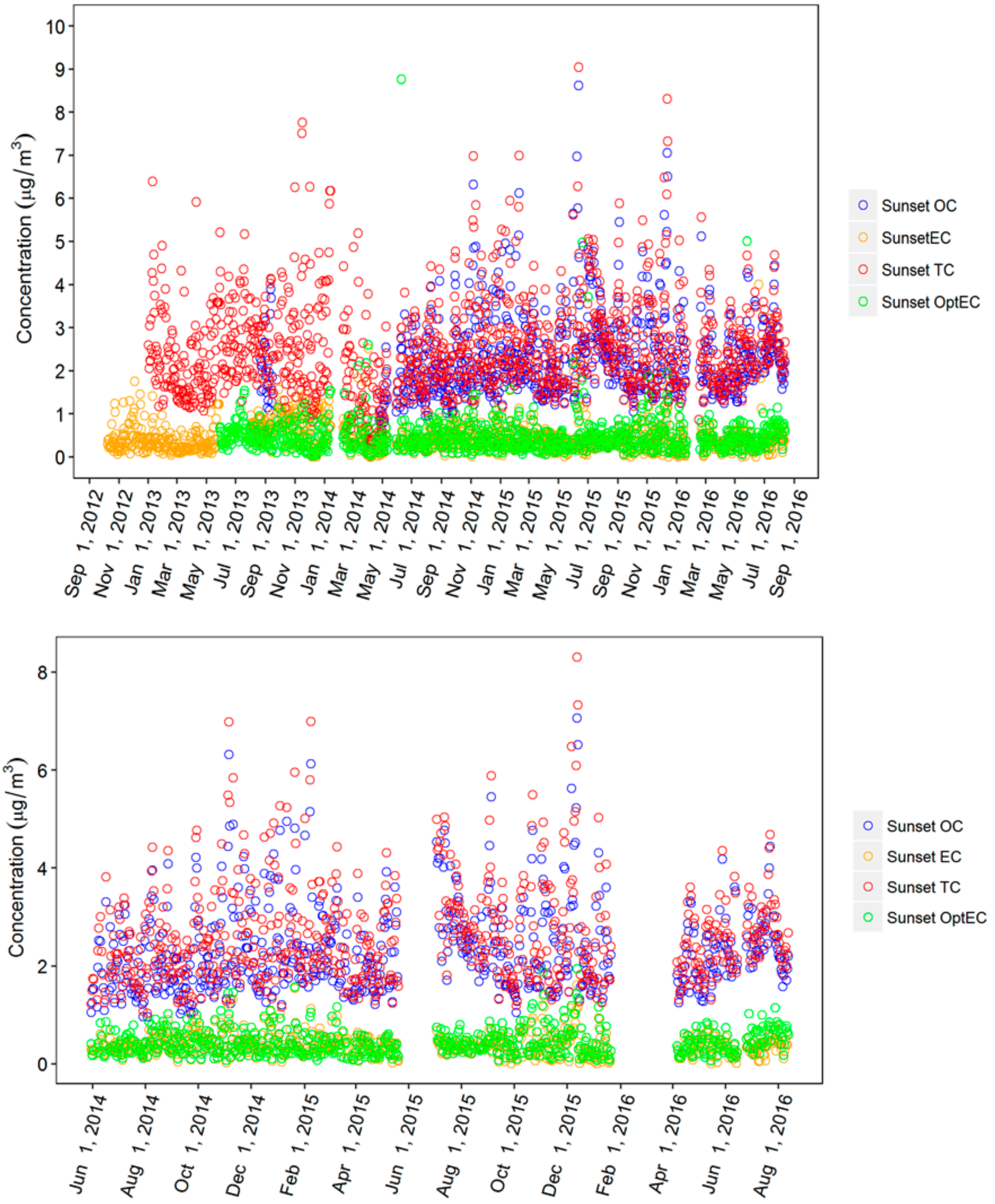

Figure S-7. Time series of Sunset data available in AQS (top) and data used in this work (bottom) for Washington, D.C. Prior to May 2014, OC was not reported, so no data were included here for analysis. Data in June 2015 and February–March 2016 were also excluded from analysis because of operational issues associated with a software update in June and a heating coil malfunction at the end of January 2016, which was not fixed until the end of March 2016.

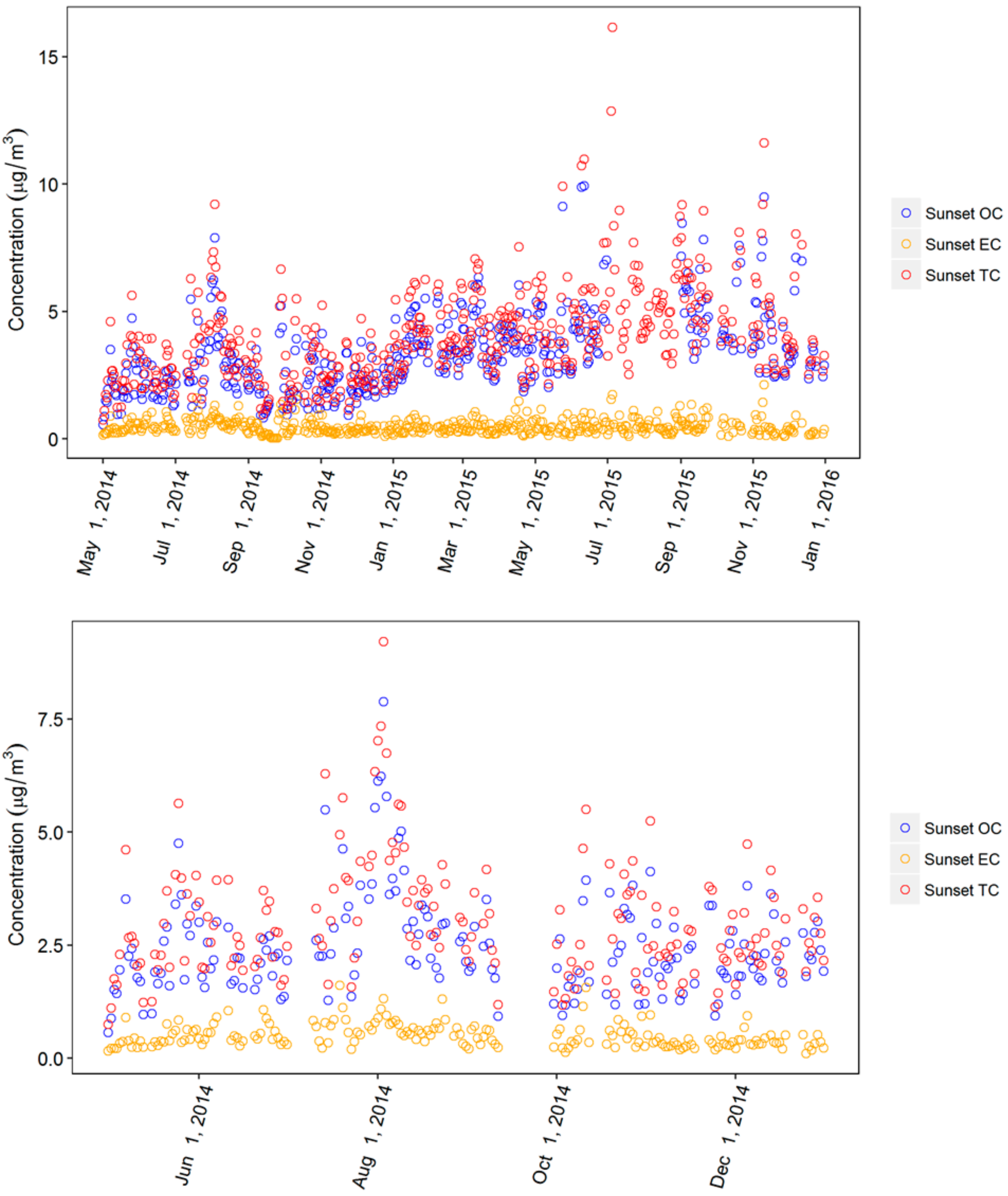

Figure S-8. Time series of Sunset data available in AQS (top) and data used in this work (bottom) for Chicago. Data after January 2015 were excluded from analysis since there is a clear gradual rise in baseline of OC due to degradation of the NDIR.

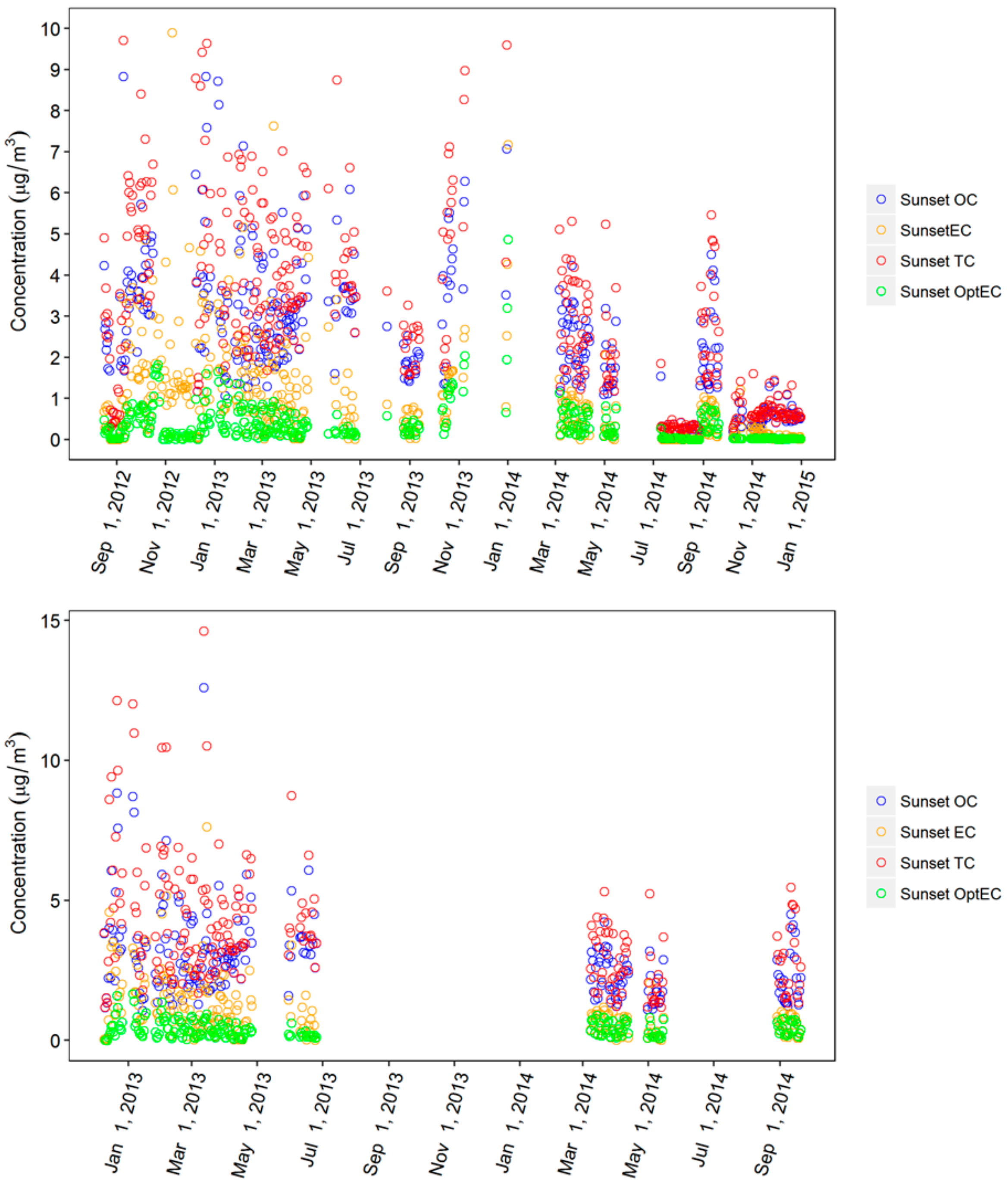

Figure S-9. Time series of Sunset data available in AQS (top) and data used in this work (bottom) for Las Vegas. Data were intermittent due to multiple operational issues. Only data with multiple weeks of consistent measurements were included for analysis. For example, in November 2012, OC was consistently reported as less than 0.5 μgC/m^3, and at other times the NDIR and heater coils broke multiple times, there was vandalism that incapacitated the shelter air conditioning unit, and instrument software was not routinely updated.

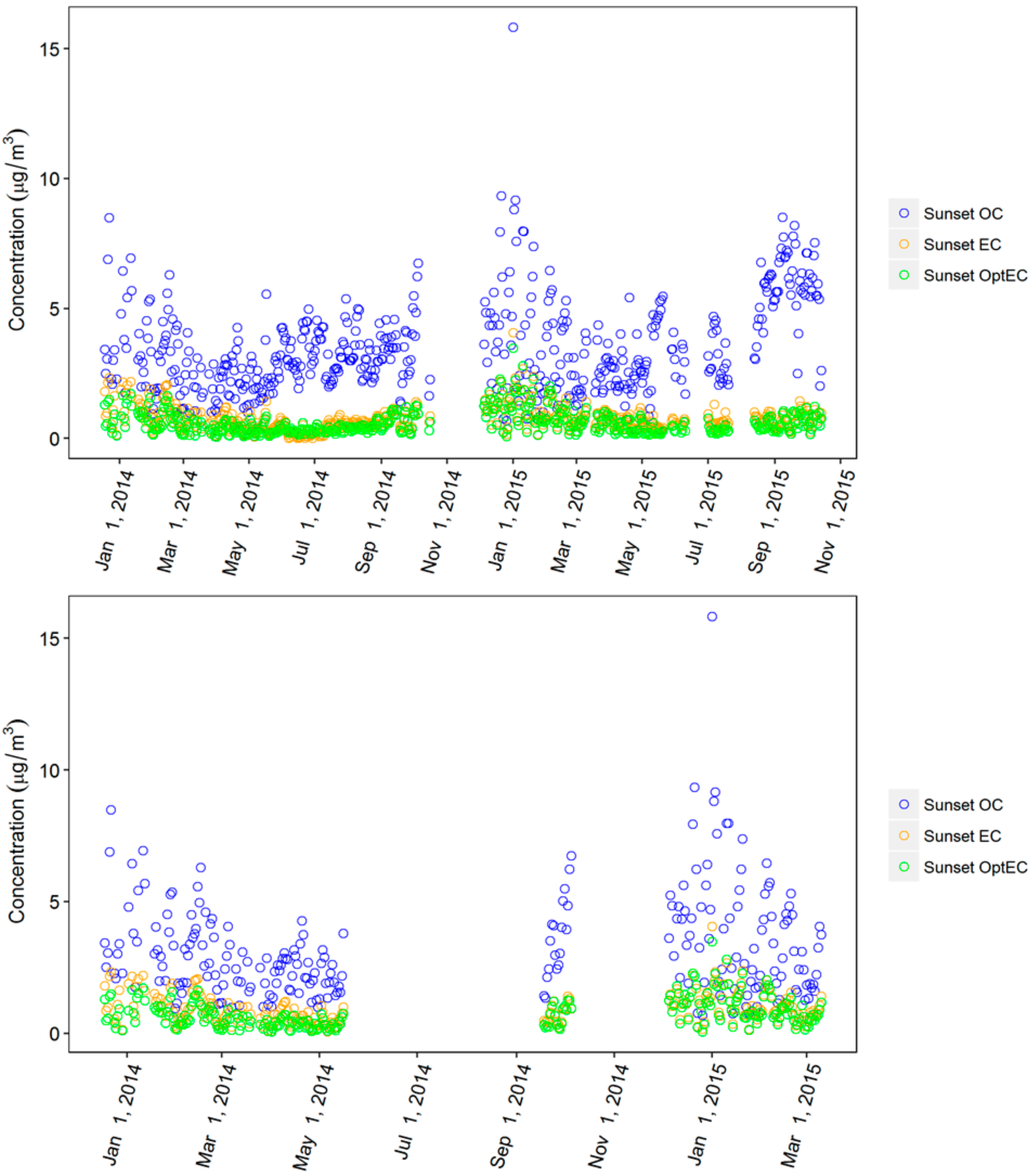

Figure S-10. Time series of Sunset data available in AQS (top) and data used in this work (bottom) for Rubidoux. In May 2014, there was a clear shift in OC upward and a shift of EC downward, and these data were excluded from analysis. During these periods, operators found leaks in the sampling line and the oven was replaced twice.

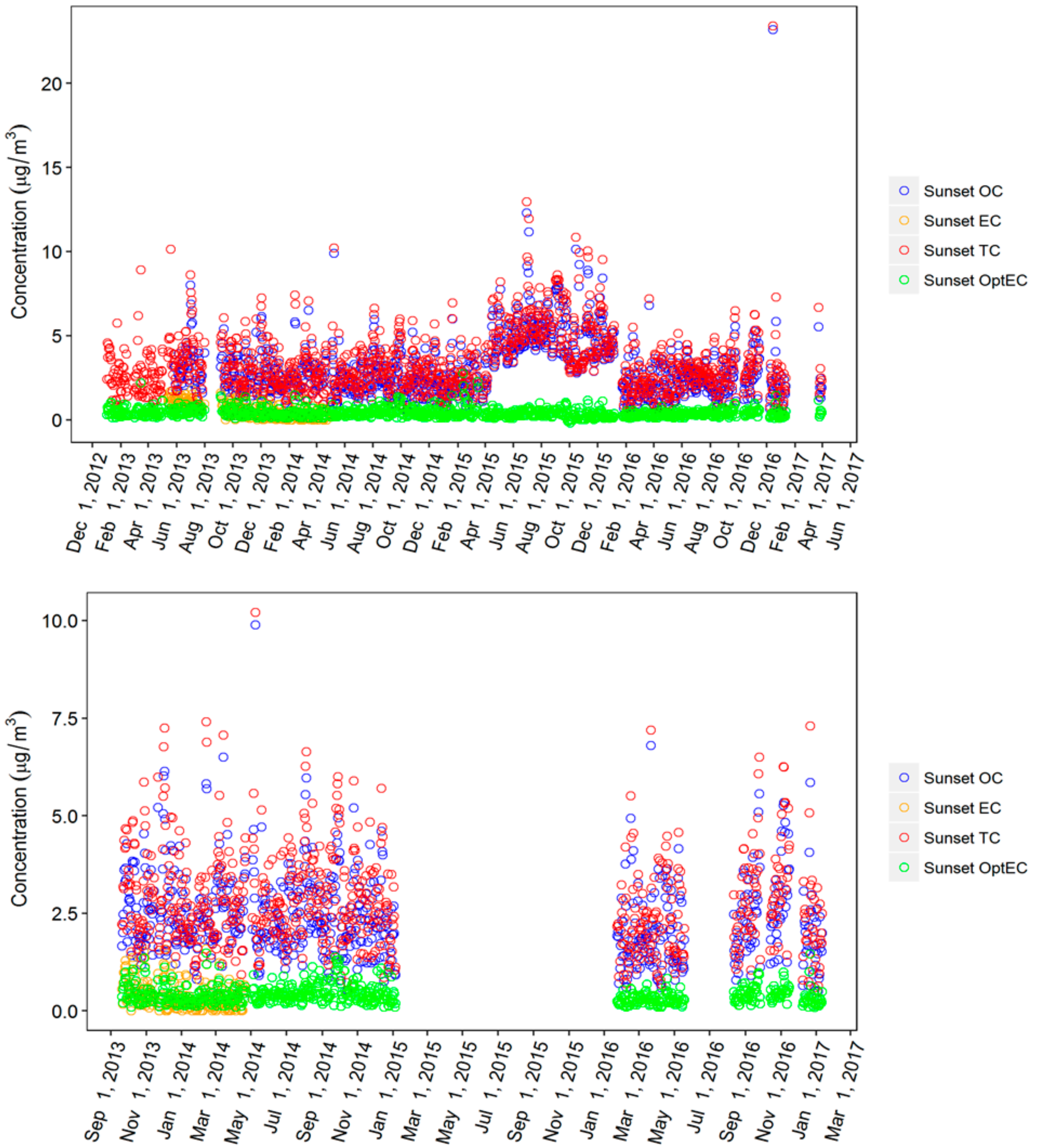

Figure S-11. Time series of Sunset data available in AQS (top) and data used in this work (bottom) for St. Louis. Data prior to September 2013 were excluded from analysis as this was a "warm-up" period when operations were getting settled. There was a sudden shift in OC concentrations starting in January 2015 when the filter was stuck and new calibration calculations were put into place. During March 2015–January 2016, operators suspected contamination, adjusted the thermocouple, and installed a new photodetector. However, data did not return to "normal" until after the oven was replaced in January 2016. There were additional issues with keeping the flow steady in June–July 2016.

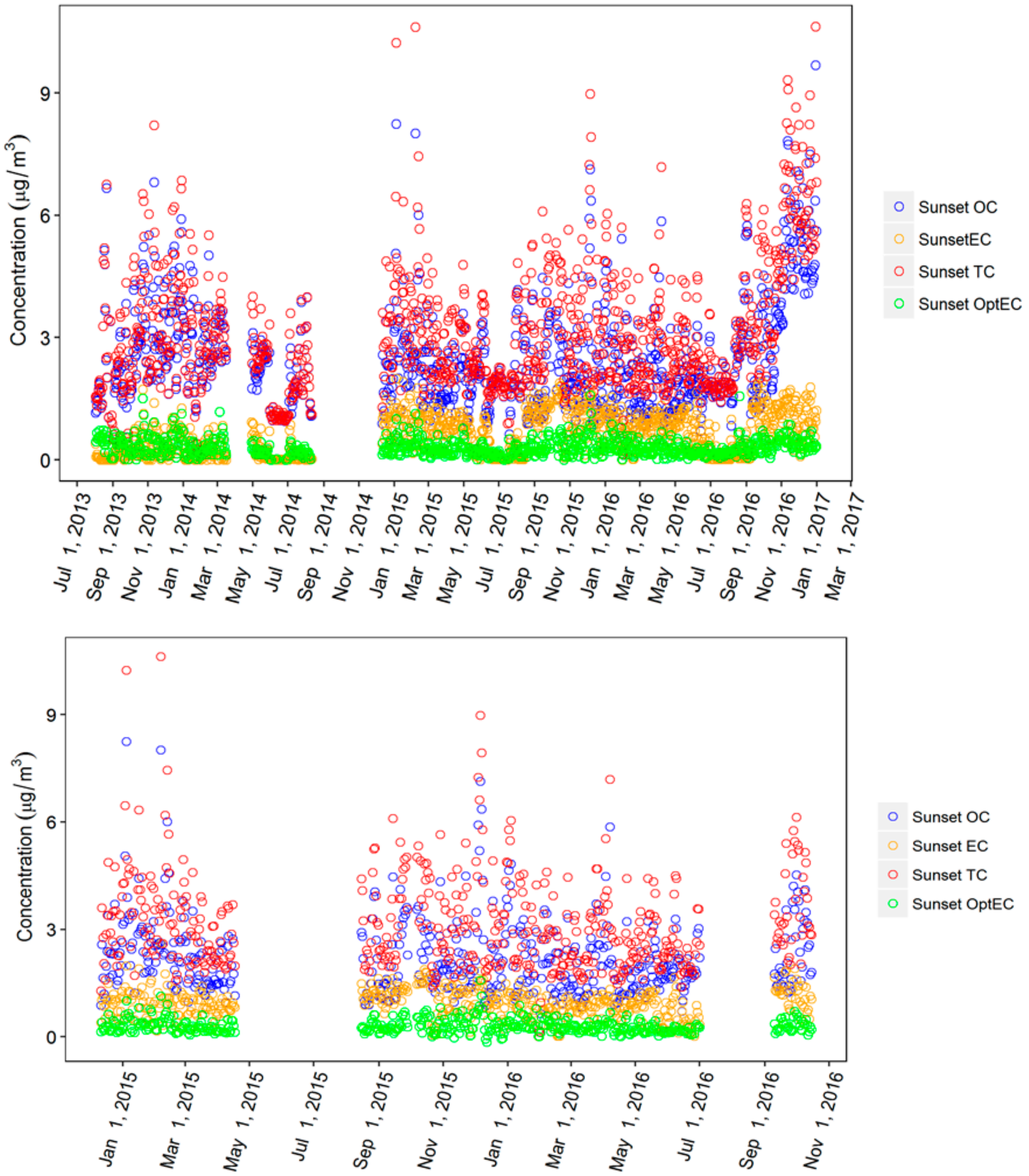

Figure S-12. Time series of Sunset data available in AQS (top) and data used in this work (bottom) for Houston. Data prior to December 2014 were excluded since older software was used to determine OC/EC and OptEC, the NDIR malfunctioned and was replaced twice, the oven thermocouple malfunctioned and was replaced, there were leaks, and the instrument was sent back to Sunset twice for maintenance. Data during May–August 2015 and in July–August 2016 had an unusual shift in OC, and EC was near zero, which were not seen in collocated measurements, and which occurred when there were leaks in the sampling line.

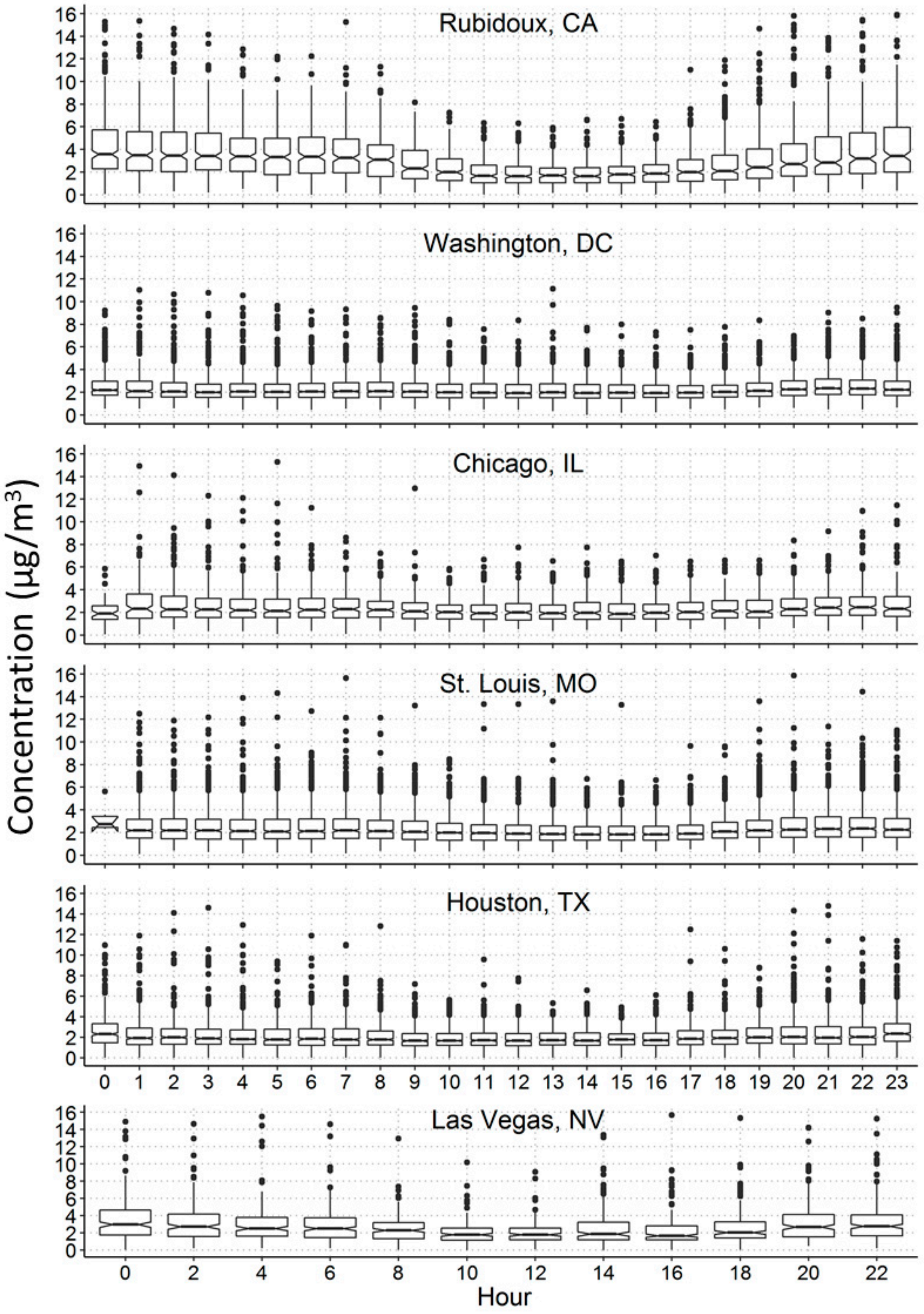

Figure S-13. Box plots of hourly Sunset OC by site.

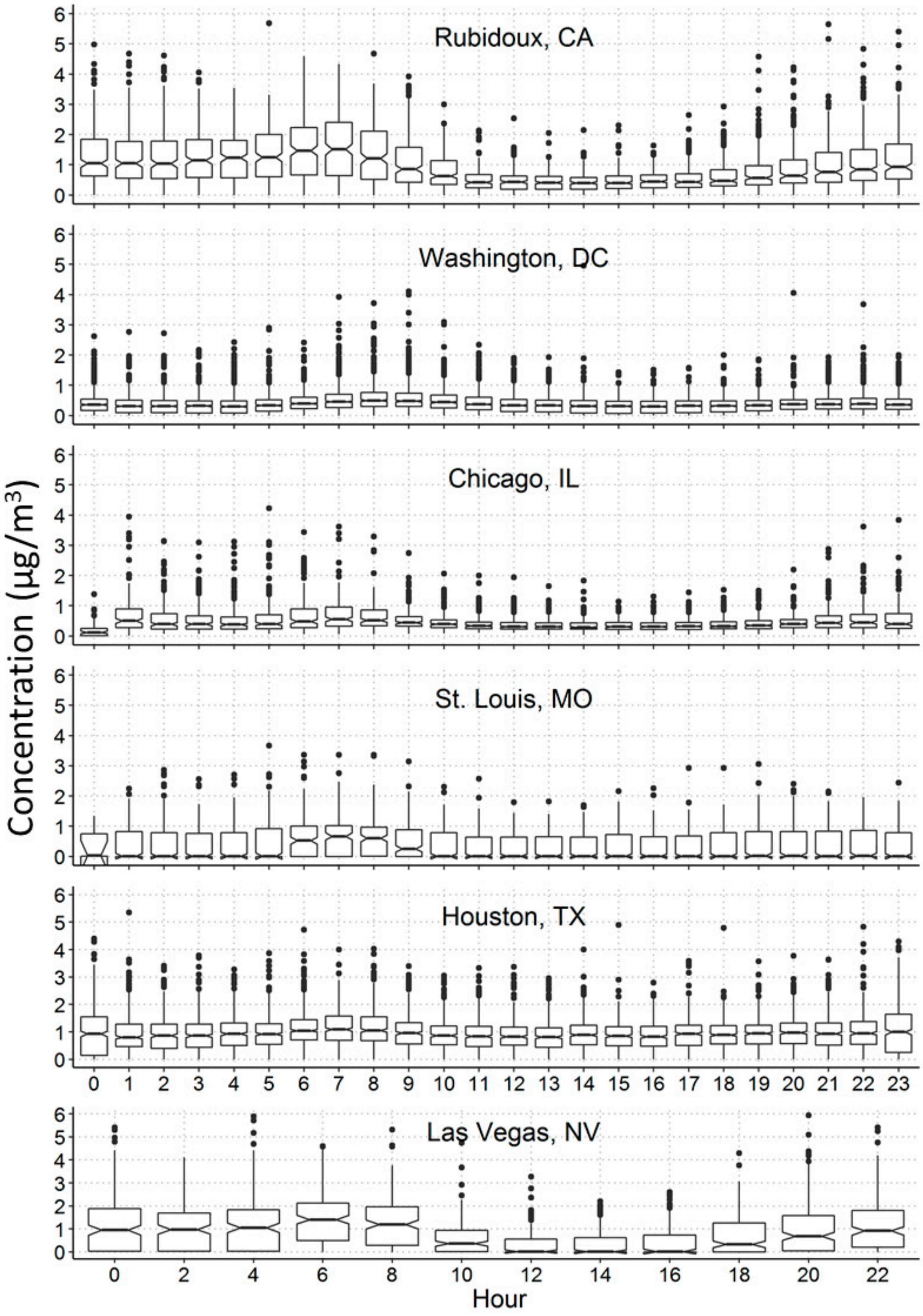

Figure S-14. Box plots of hourly Sunset EC by site.

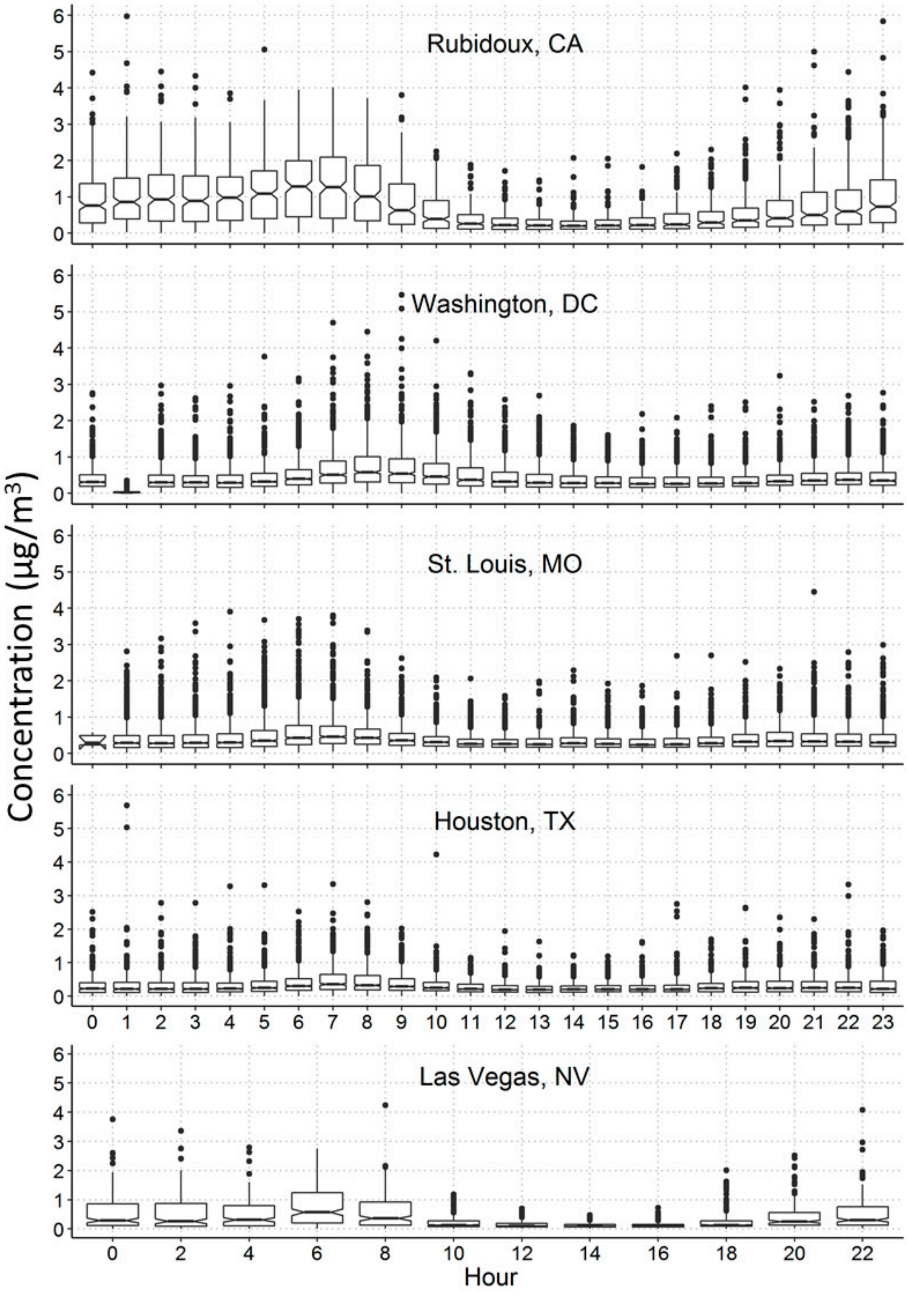

Figure S-15. Box plots of hourly Sunset OptEC by site.

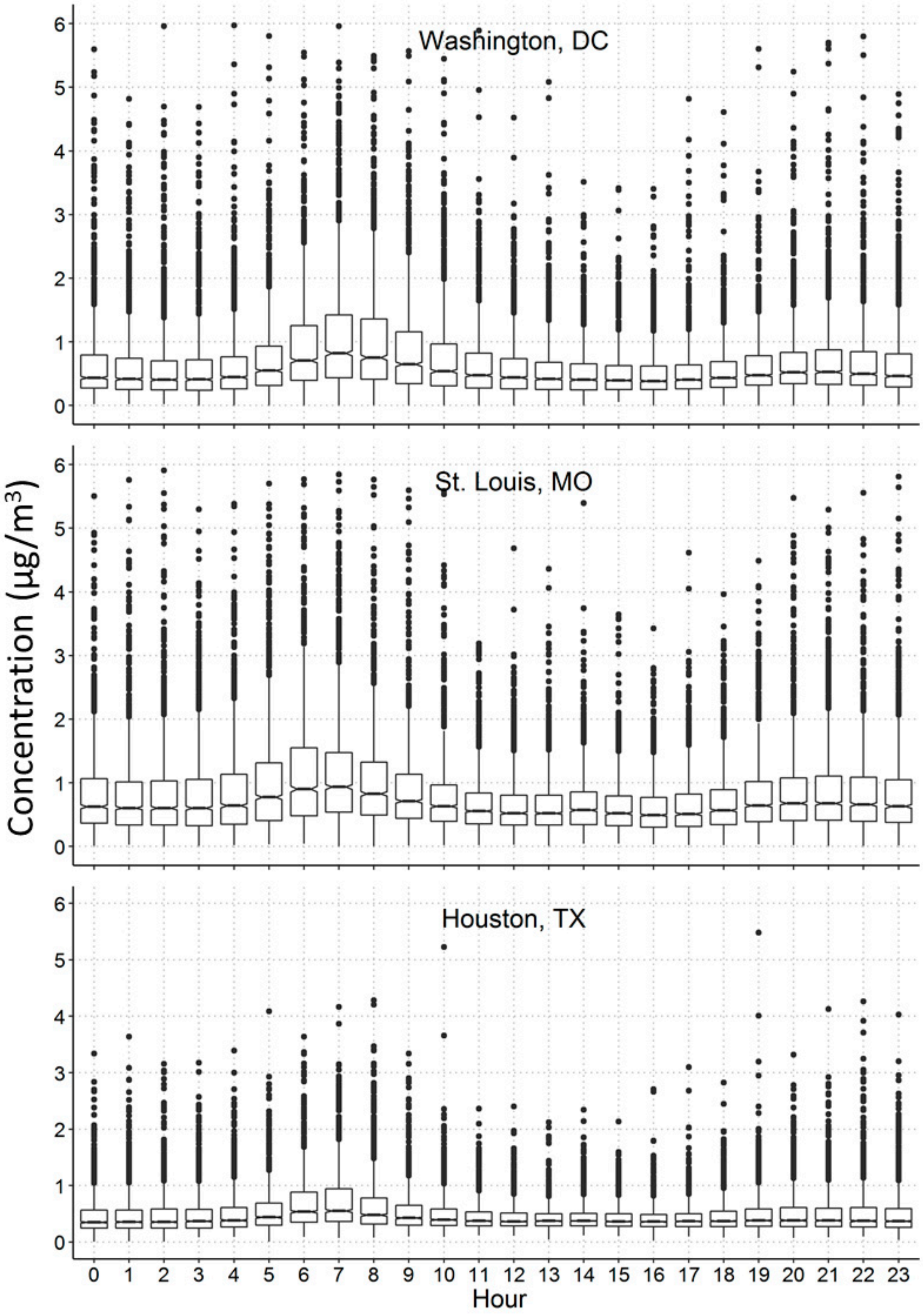

Figure S-16. Box plots of hourly Aethalometer BC by site.

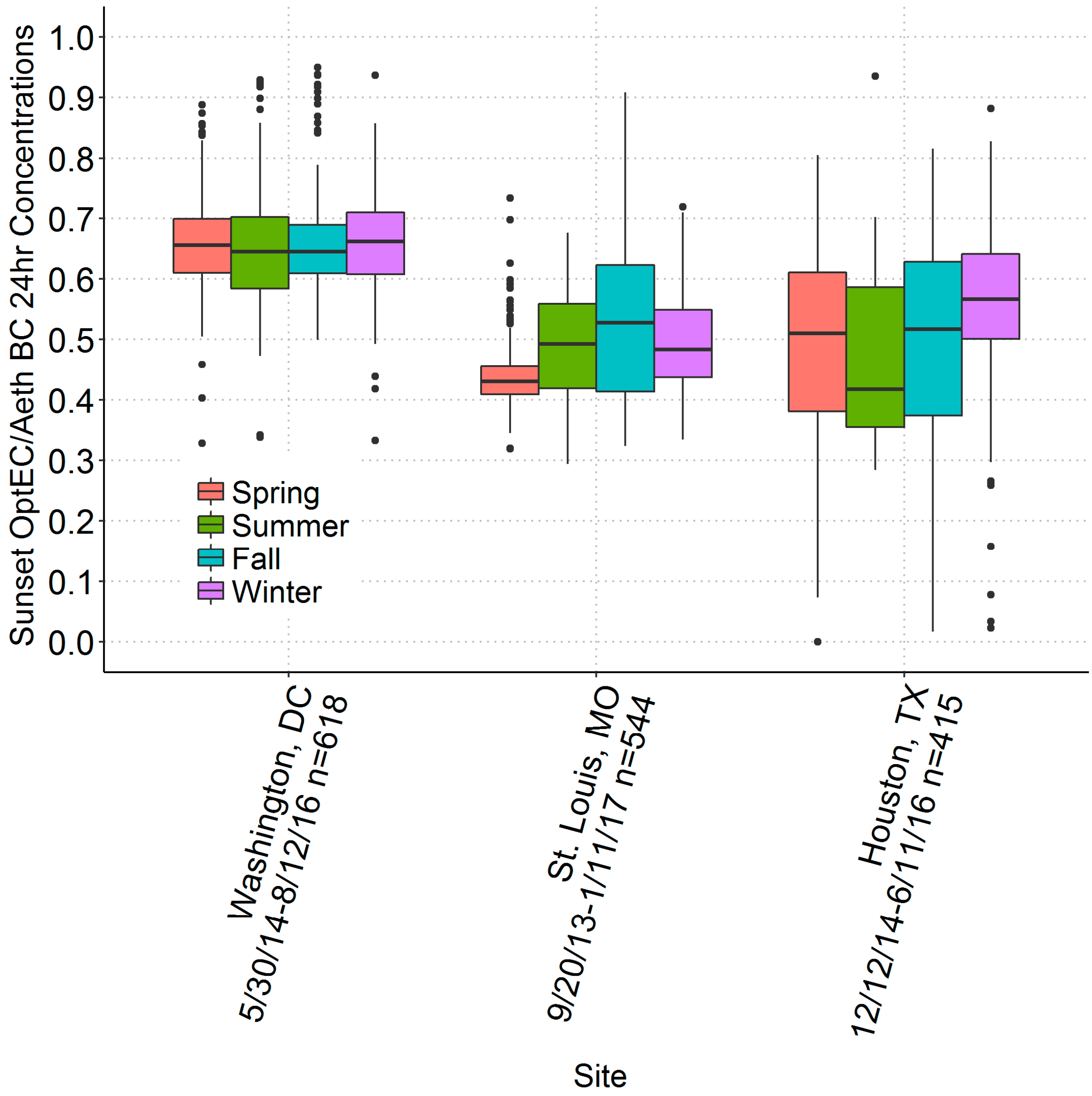

Figure S-17. Box plots of OptEC/BC ratio by season and site.
